# Analytical and behavioral characterization of 1‐hexanoyl‐LSD (1H‐LSD)

**DOI:** 10.1002/dta.3767

**Published:** 2024-07-04

**Authors:** Simon D. Brandt, Pierce V. Kavanagh, Sarah Gare, Alexander Stratford, Adam L. Halberstadt

**Affiliations:** ^1^ School of Pharmacy and Biomolecular Sciences Liverpool John Moores University Liverpool UK; ^2^ Department of Pharmacology and Therapeutics School of Medicine, Trinity Centre for Health Sciences, St. James Hospital Dublin Ireland; ^3^ Department of Chemistry, School of Physical Sciences University of Liverpool Liverpool UK; ^4^ Synex Synthetics BV Maastricht The Netherlands; ^5^ Department of Psychiatry University of California San Diego San Diego Southern California USA; ^6^ Center for Psychedelic Research University of California San Diego San Diego Southern California USA; ^7^ Research Service, VA San Diego Healthcare System San Diego Southern California USA

**Keywords:** head‐twitch response, LSD, new psychoactive substances, psychedelics

## Abstract

The development of lysergic acid diethylamide (LSD) derivatives and analogs continues to inform the design of novel receptor probes and potentially new medicines. On the other hand, a number of newly developed LSD derivatives have also emerged as recreational drugs, leading to reports of their detection in some countries. One position in the ergoline scaffold of LSD that is frequently targeted is the *N* ^1^‐position; numerous *N* ^1^‐alkylcarbonyl LSD derivatives have been reported where the acyl chain is attached to the indole nitrogen, for example, in the form of linear *n*‐alkane substituents, which represent higher homologs of the prototypical 1‐acetyl‐*N*,*N*‐diethyllysergamide (1A‐LSD, ALD‐52). In this study, 1‐hexanoyl‐LSD (1H‐LSD, SYN‐L‐027), a novel *N* ^1^‐acyl LSD derivative, was characterized analytically using standard techniques, followed by evaluation of its in vivo behavioral effects using the mouse head‐twitch response (HTR) assay in C57BL/6J mice. 1H‐LSD induced the HTR, with a median effective dose (ED_50_) of 192.4 μg/kg (equivalent to 387 nmol/kg), making it roughly equipotent to ALD‐52 when tested previously under similar conditions. Similar to other *N* ^1^‐acylated analogs, 1H‐LSD is anticipated to by hydrolyzed to LSD in vivo and acts as a prodrug. It is currently unknown whether 1H‐LSD has appeared as on the research chemical market or is being used recreationally.

## Graphical abstract

Analytical and pharmacological data were obtained from 1‐hexanoyl‐LSD (1H‐LSD). The mouse head‐twitch response revealed that 1H‐LSD induced LSD‐like effects (ED_50_ = 387 nmol/kg). The behavioral and analytical characterization of novel lysergamides aim to extend the understanding of structure–activity relationships and disseminate data for those interested in the drugs field.

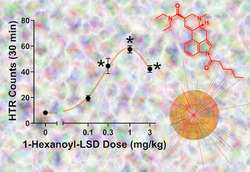

## 1 INTRODUCTION

**FIGURE 1:**
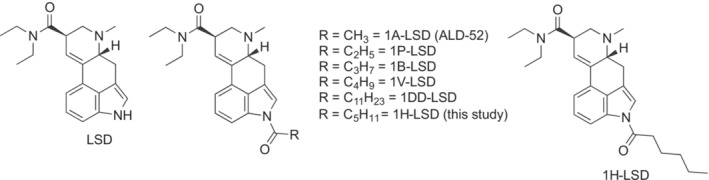
Structures of LSD and other *N* ^*1*^‐acylated derivatives. LSD, lysergic acid diethylamide.

The classical psychedelic lysergic acid diethylamide (also known as *N*,*N*‐diethyllysergamide or LSD) has remained a popular recreational substance over the past six decades. During that period, several LSD analogs have appeared as recreational drugs, including *N* ^6^‐allyl‐6‐nor‐LSD (AL‐LAD), *N* ^6^‐ethyl‐6‐nor‐LSD (ETH‐LAD), lysergic acid 2,4‐dimethylazetidide (LSZ), and *N*‐morpholinyllysergamide (LSM‐775). Various *N* ^1^‐acyl‐substituted LSD derivatives have also appeared. 1‐Propanoyl‐*N*,*N*‐diethyllysergamide (1P‐LSD, Figure 1) emerged as a recreational drug almost a decade ago.^1^, ^2^ At the time of its appearance, 1P‐LSD constituted a novel homolog of 1‐acetyl‐LSD (ALD‐52, Figure 1), a psychedelic drug synthesized at Sandoz in the 1950s.^3^ Subsequently, ALD‐52 also surfaced on the research chemicals market.^4^

At the same time, the activity and structure–activity relationships (SAR) of LSD derivatives and analogs continues to inform the design of novel pharmacological tools and potentially new medicines.^5^, ^6^ LSD and other psychedelics such as psilocybin are currently being tested as potential treatment for a range of disorders including depression, anxiety, and substance abuse. *N* ^1^‐Acylated LSD derivatives are hydrolyzed to LSD in vitro and in vivo and are believed to serve as prodrugs,^6^, ^7^, ^8^, ^9^, ^10^ meaning they may possess LSD‐like therapeutic efficacy.

In recent years, a range of other higher homologs of 1P‐LSD have been synthesized to further elucidate the pharmacological properties and SAR of lysergamides, which has sometimes paralleled their appearance as designer drugs supplied by online vendors. Examples of these linear *N* ^1^‐alkylcarbonyl LSD derivatives include 1B‐LSD,^11^, ^12^, ^13^ 1V‐LSD,^14^, ^15^ and 1DD‐LSD,^16^ though it is unknown whether the latter drug has actually appeared on the online market. As shown in Figure 1, the linear *N* ^1^‐acylated derivatives reported previously reflect C_2_–C_5_ and C_12_ carbon chain lengths, all of which induce LSD‐like behavioral effects with varying potencies using the head‐twitch response (HTR) in mice as a readout.^2^, ^6^, ^11^, ^14^, ^16^ The HTR is a rapid rotational head shaking induced by LSD in rodents that serves as a behavioral proxy for psychedelic effects in humans.

The present study explored the analytical and behavioral properties of 1‐hexanoyl‐LSD (1H‐LSD, also known as SYN‐L‐027), a novel 1P‐LSD homolog. Similar to ALD‐52 and 1P‐LSD, available information suggests that 1H‐LSD also serves as a prodrug for LSD.^10^ The patent literature indicates that 1H‐LSD induces the HTR in mice, although only one dose was tested.^10^ To fully characterize the response to 1H‐LSD and quantify its potency relative to LSD and other lysergamides, a full dose–response experiment is required. Similar to the psychedelic effects of LSD in humans, the HTR induced by LSD in mice is mediated by 5‐HT_2A_ receptor activation, making the HTR a useful readout of the psychopharmacological properties of novel lysergamides.^17^, ^18^, ^19^, ^20^

## 2 EXPERIMENTAL SECTION

### 2.1 Materials

All chemicals and solvents were of analytical or HPLC grade and were obtained from Aldrich (Dorset, UK). A powdered sample of 1H‐LSD hemitartrate (2:1) was provided by Synex Synthetics BV, Maastricht, The Netherlands.

### 2.2 Instrumentation

#### 2.2.1 Gas chromatography‐electron ionization mass spectrometry (GC‐EI‐MS)

Electron ionization mass spectra were recorded on an Agilent 5977A MSD detector (Agilent, Cheadle, UK). Temperature settings were as follows: transfer line 275°C, source 230°C, and quadrupole 150°C. The mass spectrometer settings were as follows: solvent delay 3 min; EI mode, 70 eV, and range *m/z* 28–500. Chromatographic analysis was carried out using an Agilent 7890A system (Agilent, Cheadle, UK). The carrier gas was helium at a flow rate of 1 mL/min. The injection temperature was 275°C. Separations were performed on a 30‐m × 0.25‐mm (0.25 μm film thickness) Agilent HP‐5MS column. The column temperature was programmed as follows: 100°C held for 1 min, then heated at 20°C/min to 310°C and held constant for 22.5 min (total run time 34 min). A 1‐μL solution of 1H‐LSD tartrate in acetonitrile (2 mg/mL) was injected for analysis (split: 1:25).

#### 2.2.2 Gas chromatography‐chemical ionization mass spectrometry (GC‐CI‐MS)

GC‐CI‐MS(/MS) analyses (scan range *m/z* 28–500) were carried out using a Varian 450‐GC gas chromatograph coupled to a Varian 220‐MS ion trap mass spectrometer. Samples were introduced (1 μL, 2 mg/mL, dissolved in acetonitrile) with a Varian 8400 autosampler using a CP‐1177 injector (275°C) in split mode (1:50). Data manipulation were performed with the MS Data Review function of the Workstation software, version 6.91. Transfer line, manifold, and ion trap temperatures were set at 300, 80, and 220°C, respectively. The liquid CI reagent was HPLC grade methanol. CI ionization parameters (0.4 s/scan) included the following: CI storage level 19.0 m/z; ejection amplitude 15.0 m/z; background mass 55 m/z; maximum ionization time 2000 μs; maximum reaction time 40 ms; target TIC 5000 counts. A DB‐5 ms 30 m × 0.25 mm (0.25 μm film thickness) column from Agilent (Cheadle, UK) was employed for separation. Temperature profile: The starting temperature was set at 80°C and held for 1 min. It then increased at 20°C/min to 310°C which was subsequently held constant for 22.5 min, leading to a total run time of 35 min. Tandem mass spectral recordings of the protonated molecule at m/z 422 were performed in resonant mode (excitation storage level: 155.5 m/z; excitation amplitude: 0.35 V).

#### 2.2.3 High‐performance liquid chromatography‐electrospray ionization tandem mass spectrometry (UHPLC‐QTOF‐MS/MS)

HPLC‐ESI‐QTOF‐MS was performed on a QTOF (Agilent 6,540, Cheadle, UK) instrument coupled with a 1290 Infinity II UPLC from Agilent Technologies (Cheadle, UK). Chromatographic separation was achieved on an EC C18 Poroshell 120 column (50 mm × 2.1 mm, 1.9 um particle size) from Agilent Technologies. Mobile phases A (0.1% v/v formic acid in water) and B were 0.1% v/v formic acid in acetonitrile. The elution profile was programmed as follows: *T*_min_/A:B (70:30); *T*_6_/10:90; *T*_8_/10/90); flow rate: 0.2 mL/min; column oven was at 30°C. The injection volume was 0.5 μL and 0.25 μL for MS/MS and MS, respectively. Agilent MassHunter version 08:00 was used for acquisition and analysis. The QTOF was operated in positive electrospray ionization mode, acquiring spectra in the range m/z 50–1000 (acquisition rate 1.15 spectra/s). Acquisition was performed in full scan/AutoMS/MS mode at four fixed collision energies (10–40 eV). The drying gas temperature was at 300°C with a flow rate of N_2_ at 8.0 L/min. The nebulizer gas pressure was 35 psi. Nitrogen was used as the collision gas. The voltage for the capillary was 3500 V, nozzle voltage was 1000 V and the fragmentor voltage was 100 V. Mass calibration was performed using G1969‐85000 ESI‐L low concentration tuning mix for dual ESI Jet stream source. The reference masses used to internally calibrate the QTOF were purine and HP‐0921 (121.0509 and 922.0098 Da) (Agilent Technologies). The 1H‐LSD stock solution was prepared in acetonitrile at 1.24 mg/mL, diluted 100‐fold using the mobile phase, and filtered using a 0.22‐μm nylon syringe filter.

#### 2.2.4 Nuclear magnetic resonance spectroscopy (NMR)

NMR spectra (^1^H at 600 MHz; ^13^C at 150 MHz) of the powdered sample (10 mg, 0.75 mL solvent) were recorded using a Bruker AVANCE III 600 MHz spectrometer (Bruker UK Ltd, Coventry, UK) in DMSO‐*d*_6_. Experiments were carried out at 298 K with a 5‐mm PA BBO probe with z‐gradient. Spectra were referenced to residual solvent, and assignments were supported by both 1D and 2D experiments.

### 2.3 Animal pharmacology

Male C57BL/6J mice (6–8 weeks old) were obtained from Jackson Laboratories (Bar Harbor, ME, USA) and housed up to four per cage with a reversed light‐cycle (lights on at 1900 h, off at 0700 h). Food and water were provided *ad libitum*, except during behavioral testing. Testing was conducted between 1000 and 1830 h. All animal experiments were carried out in accordance with NIH guidelines and were approved by the UCSD animal care committee. The HTR was assessed using a head‐mounted magnet and a magnetometer detection coil.^20^ Mice were anesthetized, a small incision was made in the scalp, and a small neodymium magnet was attached to the dorsal surface of the cranium using dental cement. Following a 2‐week recovery period, HTR experiments were carried out in a well‐lit room with at least 7 days between sessions to avoid carryover effects. Mice were injected IP (5 mL/kg injection volume) with vehicle (water containing 16% dimethylsulfoxide) or 1H‐LSD (0.1, 0.3, 1, or 3 mg/kg), and then, activity was recorded in a glass cylinder surrounded by a magnetometer coil for 60 min. Coil voltage was low‐pass filtered (2‐kHz cutoff frequency), amplified, digitized (20‐kHz sampling rate, 16‐bit ADC resolution), and saved to disk using a Powerlab 8/35 data acquisition system with the LabChart software ver. 8.1.16 (ADInstruments, Colorado Springs, CO, USA). To detect head twitches, events in the recordings were transformed to scalograms, deep features were extracted using the deep convolutional neural network ResNet‐50, and then, the images were classified using a support vector machine (SVM).^21^ Total head twitch counts were analyzed using a one‐way analysis of variance (ANOVA). HTR counts were also binned in 2‐min blocks and analyzed using a two‐way ANOVA (drug × time). Post hoc comparisons were made using Dunnett's test. Significance was demonstrated by surpassing an α‐level of 0.05. ED_50_ values and 95% confidence intervals were calculated using nonlinear regression. HTR counts were also binned in 5‐min blocks and analyzed using a two‐way ANOVA (drug × time). Post hoc comparisons were made using Dunnett's test. Significance was demonstrated by surpassing an *α*‐level of 0.05. ED50 values and 95% confidence intervals were calculated using nonlinear regression.

## 3 RESULTS AND DISCUSSION

### 3.1 Analytical features

**FIGURE 2:**
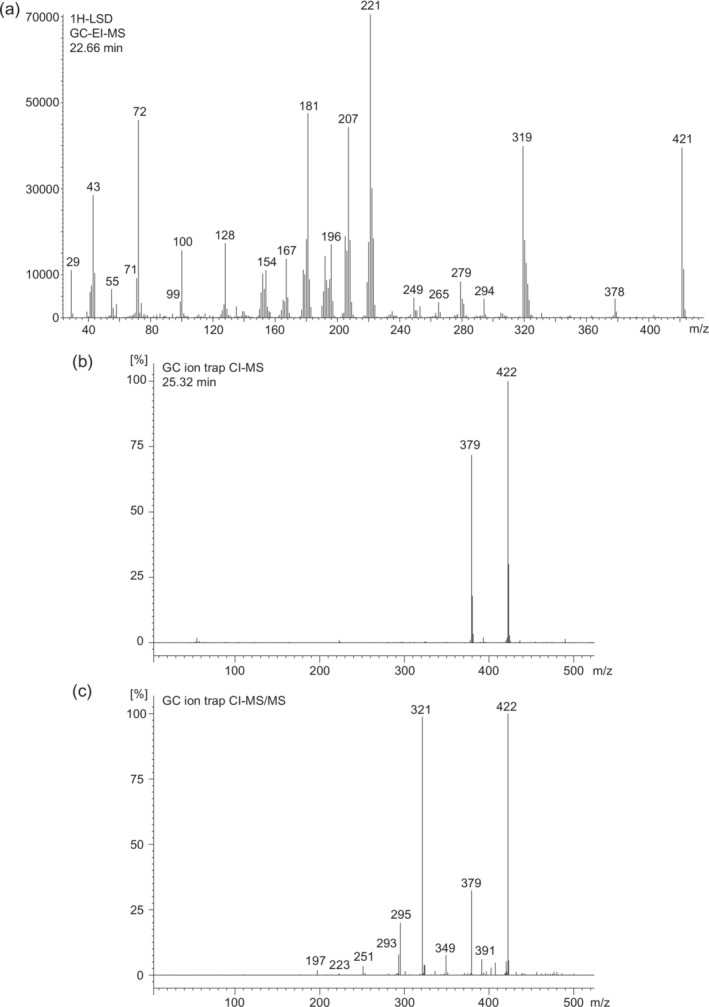
Mass spectral data recorded for 1H‐LSD. (a) Electron ionization, (b) chemical ionization single, and (c) tandem mass spectral acquisition stage. 1H‐LSD, 1‐hexanoyl‐lysergic acid diethylamide.

Figure 2(a) presents the electron ionization (EI) mass spectrum of 1H‐LSD, with proposed fragmentation pathways included as Supporting Information based on those suggested previously involving the lower 1‐acyl homologs 1P‐, 1B‐, and 1V‐LSD.^2^, ^11^, ^14^ Common ions and ion clusters commonly detected in many lysergamides have been described abundantly, so only some key ions specifically relevant for 1H‐LSD in relation to its lower homolog 1V‐LSD^14^ are discussed. In comparison to 1V‐LSD, several mass shifts of 14 u were detectable. The molecular ion increased to m/z 421 (1V‐LSD: m/z 407) followed by the loss of 43 u to give rise to the *retro*‐Diels‐Alder (RDA) fragment at m/z 378 (1V‐LSD: m/z 364). Fragment ions possibly related to the *N* ^1^‐hexanoyl group were noticeable at m/z 99, 71, 43, and 29. In 1V‐LSD, these were detected at m/z 85, 57, and 43, respectively.^14^ Corresponding to the shift of 14 u, fragment clusters reflecting 1H‐LSD were observed at m/z 319–324 (1V‐LSD: m/z 304–308).

Chemical ionization single and tandem mass spectra recorded for 1H‐LSD using an ion trap mass analyzer are shown in Figures 2(b and c). The relative abundance of protonated molecule at m/z 422 was pronounced in both cases. Under CI‐MS conditions, only the RDA fragment was recorded at m/z 379, whereas more product ions were detectable under CI‐MS/MS conditions (Figure 2(c)) and prominent product ions reflecting the presence of the 1‐acyl group included m/z 391, 379, 349, 321, and 295 (proposed fragmentation pathways included in the Supporting Information document). The implementation of both GC–MS methods also revealed the detection of hexanoic acid (data not shown), which was consistent with the *N* ^1^‐hexanoyl group. The fact that some hexanoic acid residues were also detectable by NMR (see below) suggested that it might have been related to the use of the corresponding acylation reagent during synthesis.

**FIGURE 3:**
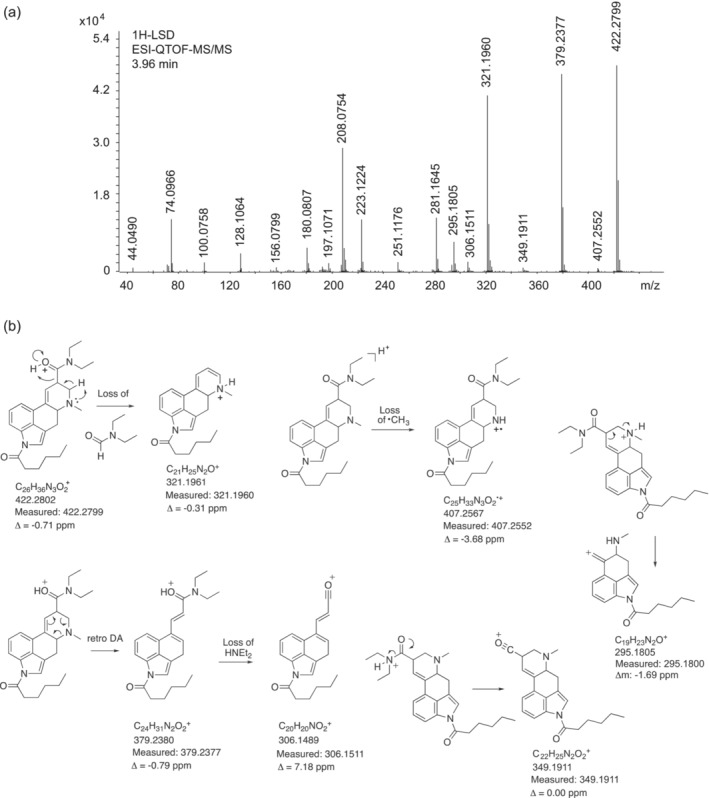
(a) Electrospray ionization QTOF tandem mass spectrum of 1H‐LSD. (b) Proposed fragmentation pathways of selected ions reflecting the presence of the 1‐hexanoyl group. 1H‐LSD, 1‐hexanoyl‐lysergic acid diethylamide; QTOF, quadrupole time of flight.

The electrospray ionization QTOF tandem mass spectrum of 1H‐LSD is shown in Figure 3(a). Examples of product ions reflecting the presence of the 1‐hexanoyl group have been proposed in Figure 3(b). Other product ions typically detected under similar conditions detectable independent of the nature of the 1‐acyl moiety have been abundantly reported previously.^2^, ^11^, ^14^ Compared to its lower homolog 1V‐LSD,^14^ the 14 u mass shift for the protonated molecule was observed at m/z 422.2799 (1V‐LSD: m/z 408.2656). Other 1‐hexanoyl related product ions included the *retro*‐Diels‐Alder fragment (RDA) at m/z 379.2377 (1V‐LSD: m/z 365.2234) and the *N* ^6^‐demethyl radical cation at m/z 407.2552 (1V‐LSD: m/z 392.2422). A neutral loss of *N*,*N*‐diethylformamide would have been consistent with m/z 321.1961 (1V‐LSD: m/z 307.1814). Other examples can be found in Figure 3(a and b).

Table 1 summarizes the nuclear magnetic resonance (NMR) spectroscopy data for 1H‐LSD, with full spectra shown as Supporting Information. The assignments were supported by HSQC and HMBC experiments and were essentially consistent with the lower homologs reported previously.^2^, ^11^, ^14^ For 1H‐LSD, the four methylene protons found in the 1‐acyl group were reflected by three resonances in the proton NMR spectrum with H‐24 and H‐25 coalescing (1.39–1.31 ppm), whereas 1V‐LSD presented three separate methylene resonances for the three sets of methylene groups. For comparison, stacked proton and carbon NMR spectra recorded from 1V‐LSD and 1H‐LSD have been included as Supporting Information. The proton integral for tartaric acid at 4.26 ppm (Table 1) was estimated to represent ~6%. GC–MS analysis revealed the presence of hexanoic acid, which was consistent with its detection by NMR. A sample of hexanoic acid was subjected to NMR analysis and included as Supporting Information for comparison.

**TABLE 1:** ^1^H and ^13^C NMR data for 1H‐LSD tartrate in DMSO‐*d*_6_ at 600/150 MHz.

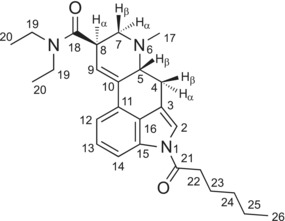		
No.	^13^C [δ/ppm]	^1^H [δ/ppm]
2	120.08	7.60 (d, *J* = 1.8 Hz, 1 H)
3	115.97	–
4	26.04	2.49–2.44 (m, 1 H) *partially overlapping with H‐17 3.49 (dd, *J* = 15.3, 5.4 Hz, 1 H)
5	61.77	3.11–3.08 (m, 1 H)
6	–	–
7	55.26	3.02 (dd, *J* = 11.2, 4.9 Hz, 1 H) 2.63 (t, *J* = 10.8 Hz, 1 H)
8	38.83	3.83–3.82 (m, 1 H)
9	121.81	6.35 (s, 1 H)
10	133.39	–
11	127.73	–
12	116.59	7.35 (d, *J* = 7.4 Hz, 1H)
13	125.93	7.30 (t, *J* = 7.8 Hz, 1H)
14	114.86	8.01 (d, *J* = 7.8 Hz, 1H)
15	133.15	–
16	127.55	–
17	43.06	2.50 (s, 3 H) *overlapping with solvent and partially overlapping with H‐4 (1H)
18	170.32	–
19	41.58	3.44 (q, *J* = 7.1 Hz, 2 H)
19	39.71	3.31 (AB qq, *J* = 13.0, 7.0 Hz, 2 H)
20	14.82	1.18 (t, *J* = 7.1 Hz, 3 H)
20	13.06	1.06 (t, *J* = 7.1 Hz, 3 H)
21	171.84	–
22	34.66	2.97 (t, *J* = 7.3 Hz, 2 H)
23	23.86	1.69 (p, *J* = 7.3 Hz, 2 H)
24	30.71	1.39–1.31 (m, 2 H)
25	21.93	1.39–1.31 (m, 2 H)
26	13.85	0.89 (t, *J* = 7.1 Hz, 3 H)
TA	173.23	–
TA	72.05	4.26 (s, ~1.8 H)

Abbreviations: TA, tartaric acid; NMR, nuclear magnetic resonance spectroscopy; 1H‐LSD, 1‐hexanoyl‐lysergic acid diethylamide; DMSO, dimethyl sulfoxide.

### 3.2 Head twitch response (HTR)

**FIGURE 4:**
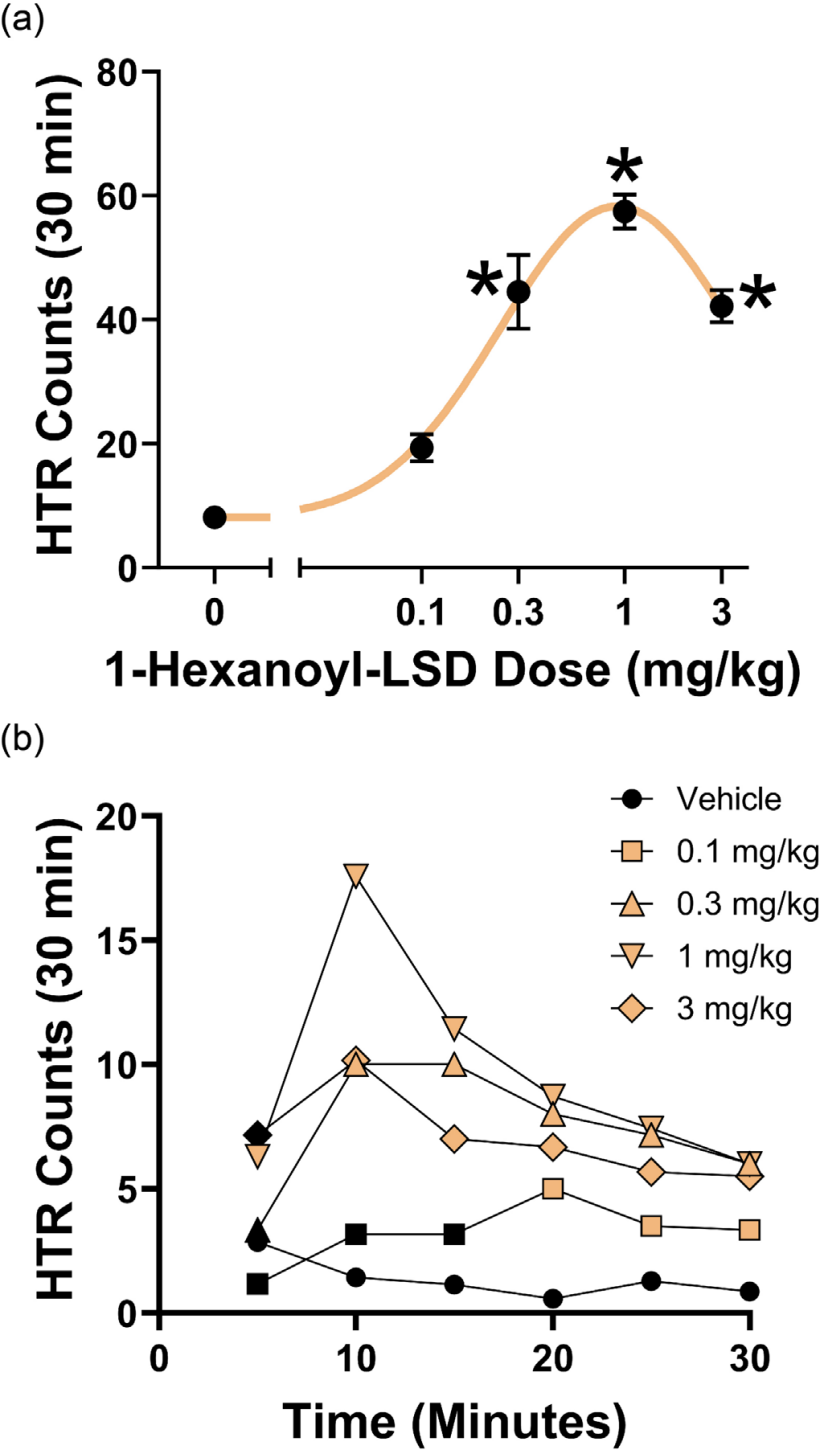
(a) Effect of 1H‐LSD on the head twitch response. Data are presented as group means ± SEM for the entire 60‐min test session. *p < 0.0001, significant difference from vehicle control group (Tukey's test). (b) Time course of the head twitch response induced by 1H‐LSD. Data are presented as group means during 5‐min time blocks. The time blocks where there were significant differences from the vehicle control group are identified using colored symbols, *p* < 0.05 (Dunnett's test). 1H‐LSD, 1‐hexanoyl‐lysergic acid diethylamide; SEM, standard error of the mean.

1H‐LSD was tested in the mouse HTR assay to determine whether it produces LSD‐like behavioral effects. As shown in Figure 4(a), 1H‐LSD induced a dose‐dependent increase in HTR counts (*W*[4,12.79] = 69.39, *P* < 0.0001). Similar to LSD and other psychedelic drugs,^17^, ^22^ 1H‐LSD induced the HTR with an inverted U‐shaped dose–response function. The median effective dose (ED_50_) for 1H‐LSD was 192.4 (145.4–254.7) μg/kg, which is equivalent to 387 (293–513) nmol/kg. The activity of 1H‐LSD is roughly equivalent to the potency of ALD‐52 (ED_50_ = 297.2 nmol/kg), 1P‐LSD (ED_50_ = 349.6 nmol/kg), 1V‐LSD (ED_50_ = 373 nmol/kg), and 1cP‐LSD (ED_50_ = 430 nmol/kg) when they were tested under similar experimental conditions.^2^, ^6^, ^14^, ^23^ Lysergamides containing an *N* ^1^‐substituent act as weak 5‐HT_2A_ receptor partial agonists, but are hydrolyzed to LSD both in vivo and in vitro and hence are believed to serve as prodrugs for LSD. Similar to other *N* ^1^‐substituted derivatives 1H‐LSD is also likely to be hydrolyzed to LSD and act as a prodrug.^10^

When tested in HTR, the maximal response to LSD occurs 5–10 min after IP dosing.^20^ When HTR data for 1H‐LSD were binned in 5‐min blocks (Figure 4(b)), the response showed a similar time‐course (dose × time: *F*[20,135] = 5.69, *P* < 0.0001), which indicated that the response to 1H‐LSD generally matched the time‐course of other *N* ^1^‐alkylcarbonyl‐substituted LSD derivatives such as 1P‐LSD^2^ and 1B‐LSD.^11^ One exception appeared to be 1DD‐LSD, which produced a response that peaked 35 min post‐dose.^16^

In previous studies, LSD induced the HTR with an ED_50_ of 132.8 nmol/kg,^20^ making 1H‐LSD about one‐third as potent as LSD in mice. However, the extent to which the relative potencies of LSD and 1H‐LSD in mice can be extrapolated to humans is unclear. For LSD and a large series of structurally diverse psychedelic drugs, there is a robust correlation (*r* = 0.9448) between their potencies (ED_50_ values) in the HTR assay mice and their potencies as psychedelic drugs in humans.^17^ However, if *N* ^1^‐substituted lysergamides act as prodrugs; then, the relationship between psychedelic drug potencies in mice and humans may not extend to *N* ^1^‐substituted lysergamides because there may be considerable cross‐species variations in their potency due to potential differences in the identity, expression level, and tissue distribution of the enzymes responsible for the hydrolysis to LSD.

## 4 CONCLUSION

The development of novel lysergamides and their corresponding prodrugs containing *N* ^1^‐acyl substituents could potentially facilitate further studies in the psychoactive drugs field. Therefore, the analytical data for 1H‐LSD collected in this study may facilitate multidisciplinary studies of this and other psychoactive lysergamides. The HTR data confirmed that 1H‐LSD has LSD‐like behavioral activity in vivo, indicating that it may act as a psychedelic drug. Ultimately, further testing is required to evaluate the pharmacodynamics and pharmacokinetics of 1H‐LSD, as well as to characterize the qualitative nature of its effects in animals and humans and its abuse potential.

## Supporting information


**Data S1.** Supporting Information.
