# Supplementary material for: Analytical and behavioral characterization of 1‐hexanoyl‐LSD (1H‐LSD)
Source: Drug Test Anal. 2024 Jul 4;17(4):561–9. doi: 10.1002/dta.3767 (PMC11994379; doi:10.1002/dta.3767)
Supplement: Supplementary file 1 — Data S1. Supporting Information. [file DTA-17-561-s001.pdf]

Analytical and behavioral characterization of 1-hexanoyl-LSD (1H-LSD)

Simon D. Brandt,[1*] Pierce V. Kavanagh,[2] Sarah Gare,[3] Alexander Stratford,[4] Adam L. Halberstadt [5,6,7]

[1] *School of Pharmacy and Biomolecular Sciences, Liverpool John Moores University, Byrom Street, Liverpool, L3 3AF, UK*

[2] *Department of Pharmacology and Therapeutics, School of Medicine, Trinity Centre for Health Sciences, St. James Hospital, Dublin 8, Ireland*

[3] *Department of Chemistry, School of Physical Sciences, University of Liverpool, Crown Street, Liverpool, L69 7ZD, UK*

[4] *Synex Synthetics BV, Maastricht, The Netherlands*

[5] *Department of Psychiatry, University of California San Diego, La Jolla, USA*

[6] *Center for Psychedelic Research, University of California San Diego, La Jolla, USA*

[7] *Research Service, VA San Diego Healthcare System, San Diego, USA*

* Correspondence to: Simon D. Brandt, School of Pharmacy and Biomolecular Sciences, Liverpool John Moores University, Byrom Street, Liverpool, L3 3AF, UK. E-Mail: s.brandt@ljmu.ac.uk

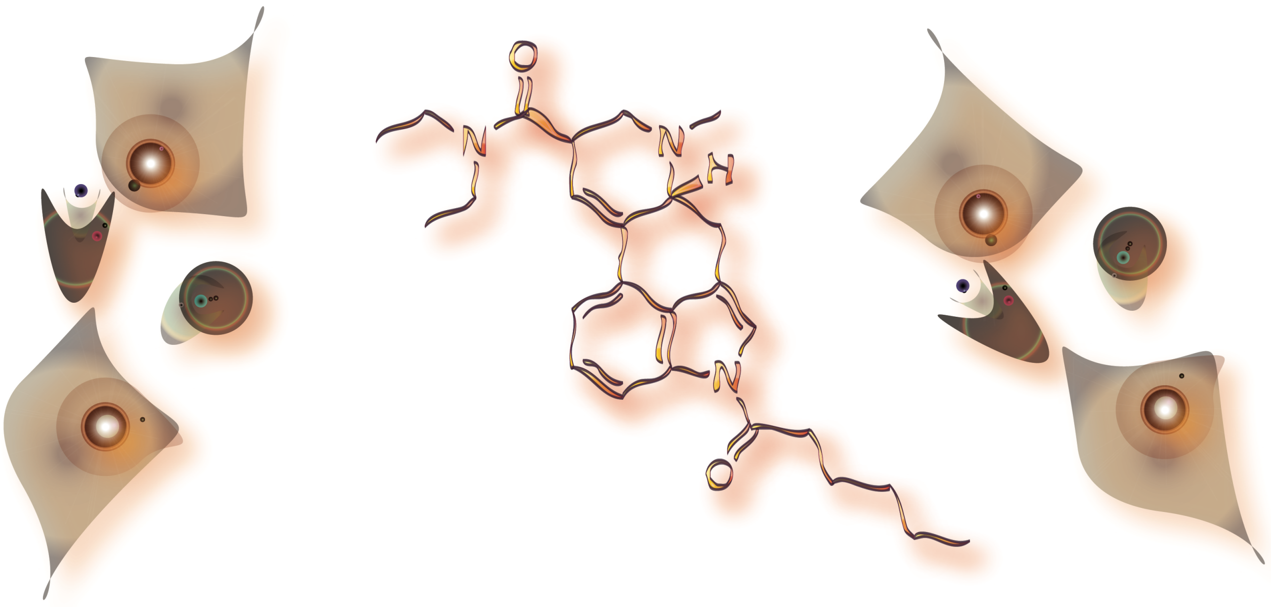

**Content**	**Page**
Proposed EI-MS fragmentation pathways (1H-LSD)	S3–S4
Proposed CI-MS/MS fragmentation pathways for 1H-LSD	S5
Attenuated total reflection-infrared spectroscopy (ATR-IR) spectra (salt vs. base)	S6–S7
LC-UV conditions and data	S8
^{1}H NMR	S9–S12
^{1}H/^{1}H COSY	S13–S16
^{13}C NMR	S17
^{13}C DEPTQ	S18
^{1}H/^{13}C HSQC	S19–S24
^{1}H/^{13}C HMBC	S25–S32
^{1}H NMR comparison 1V-LSD vs. 1H-LSD	S33–S34
^{13}C NMR comparison 1V-LSD vs. 1H-LSD	S35–S36
^{1}H NMR comparison 1H-LSD vs. hexanoic acid (partial spectrum)	S37
^{13}C NMR comparison 1H-LSD vs. hexanoic acid	S38–S39

Proposed EI-MS fragmentation pathways for 1H-LSD

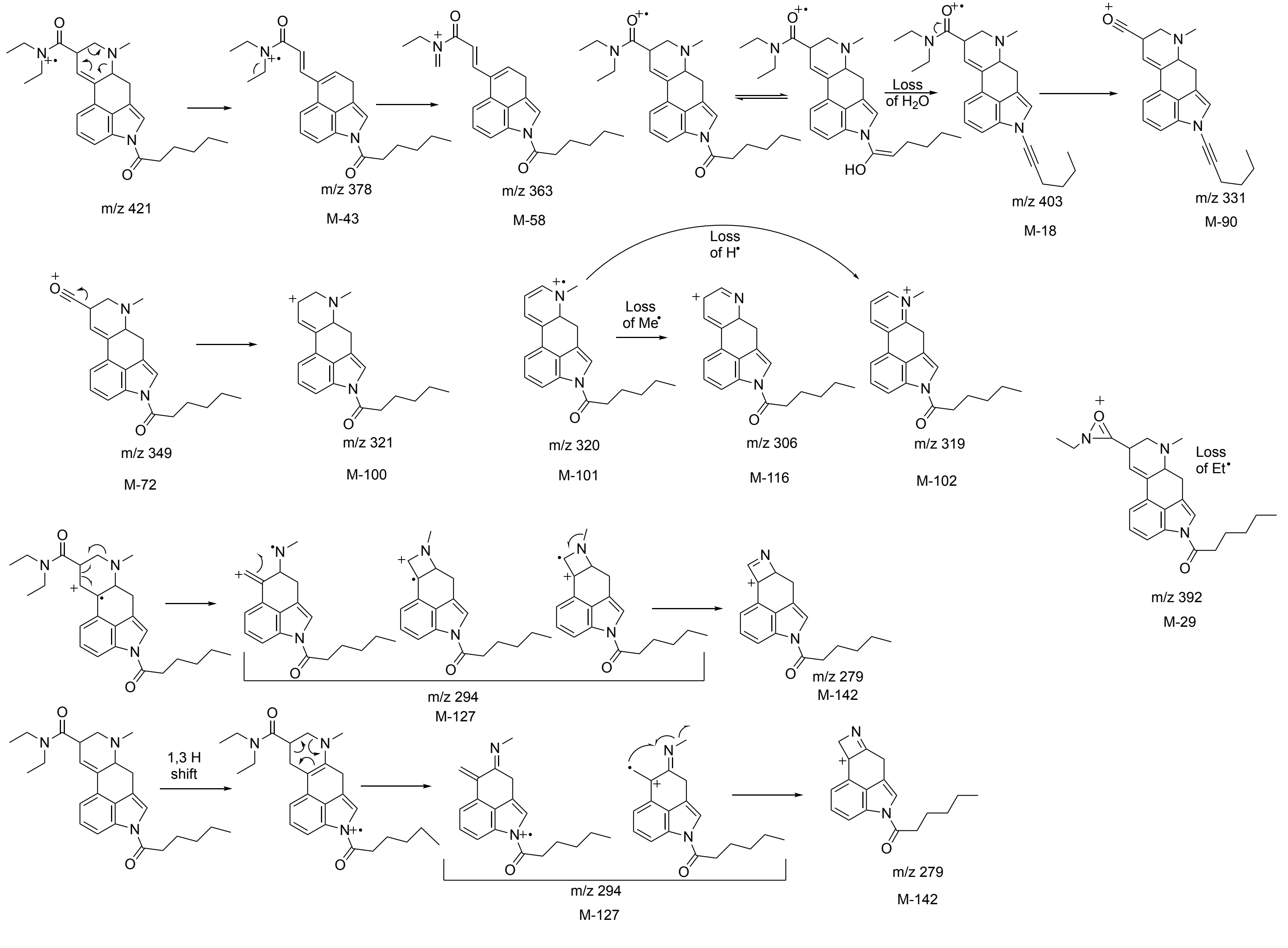

Proposed EI-MS fragmentation pathways for 1H-LSD

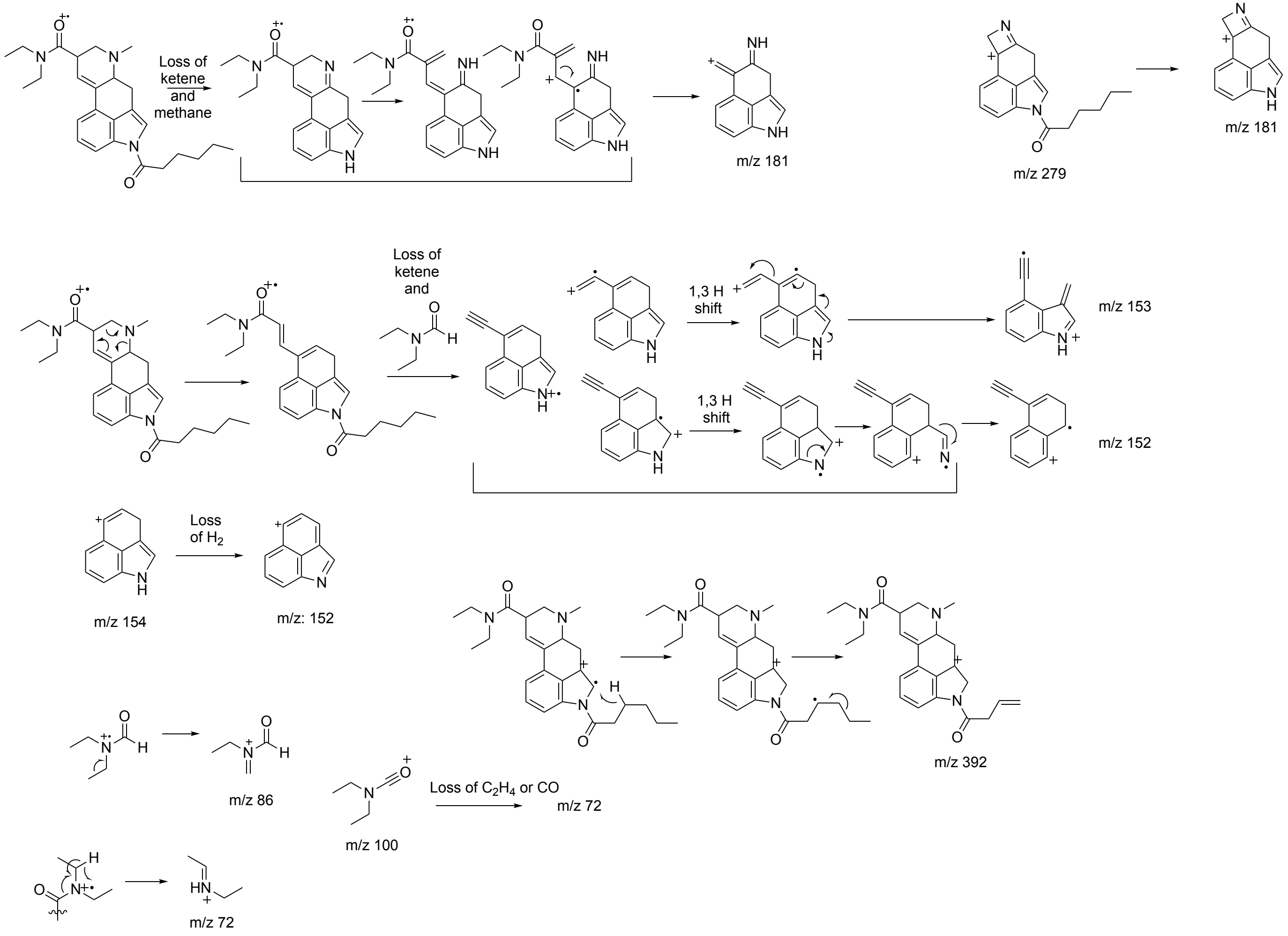

Proposed CI-MS/MS fragmentation pathways for 1H-LSD

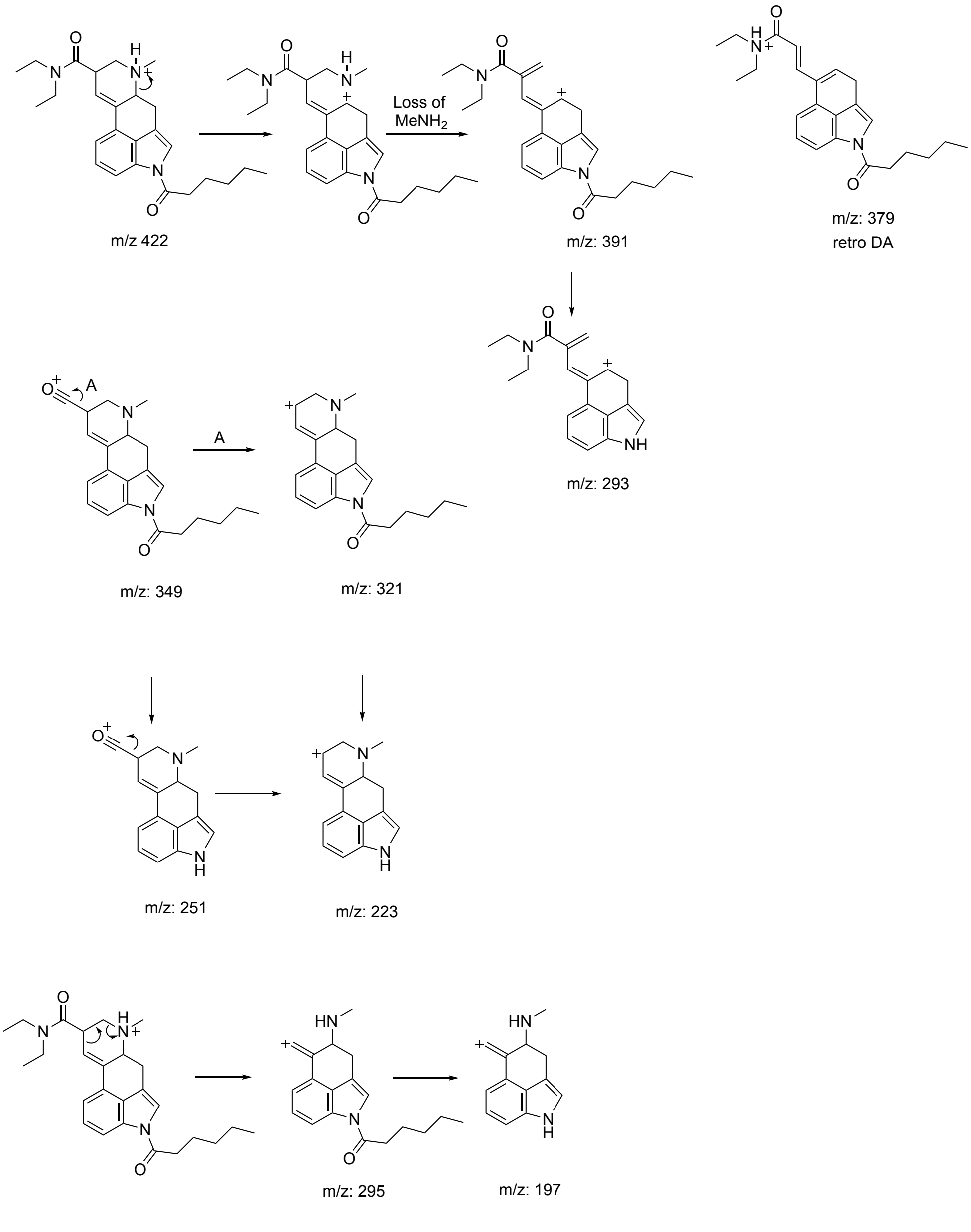

Partial ATR-IR spectra (salt vs. base)

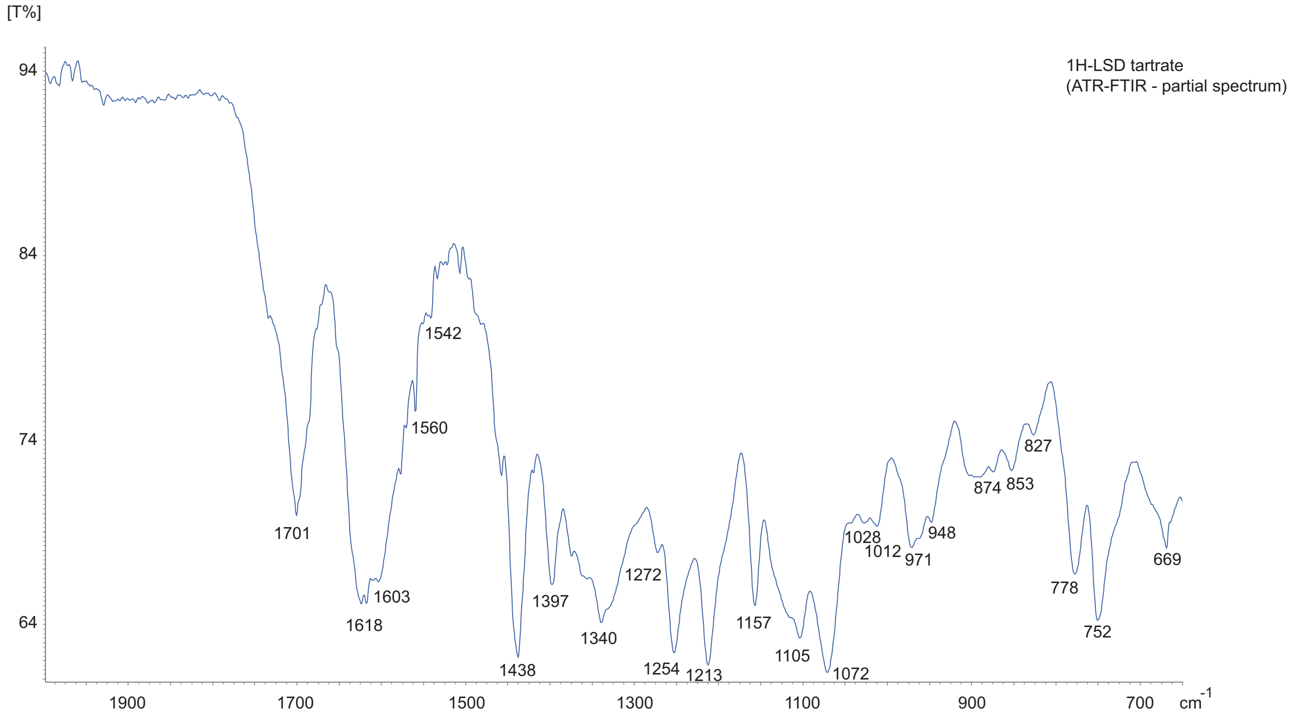

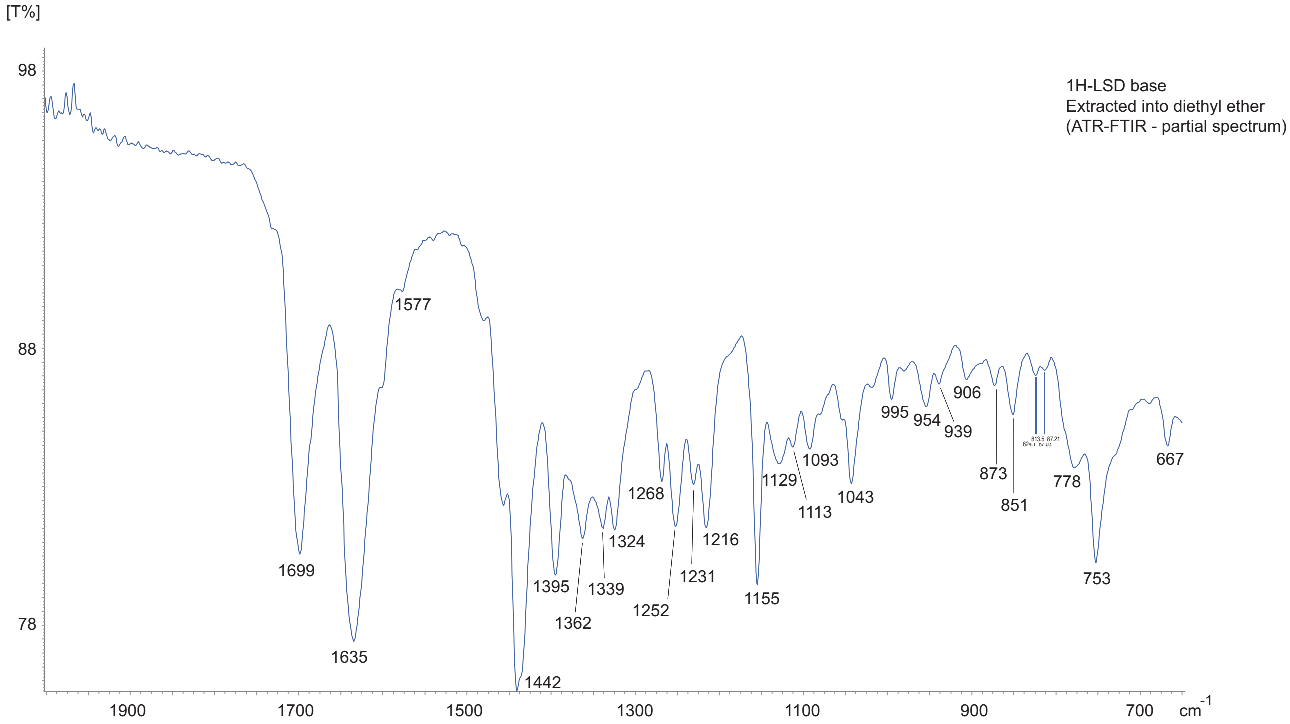

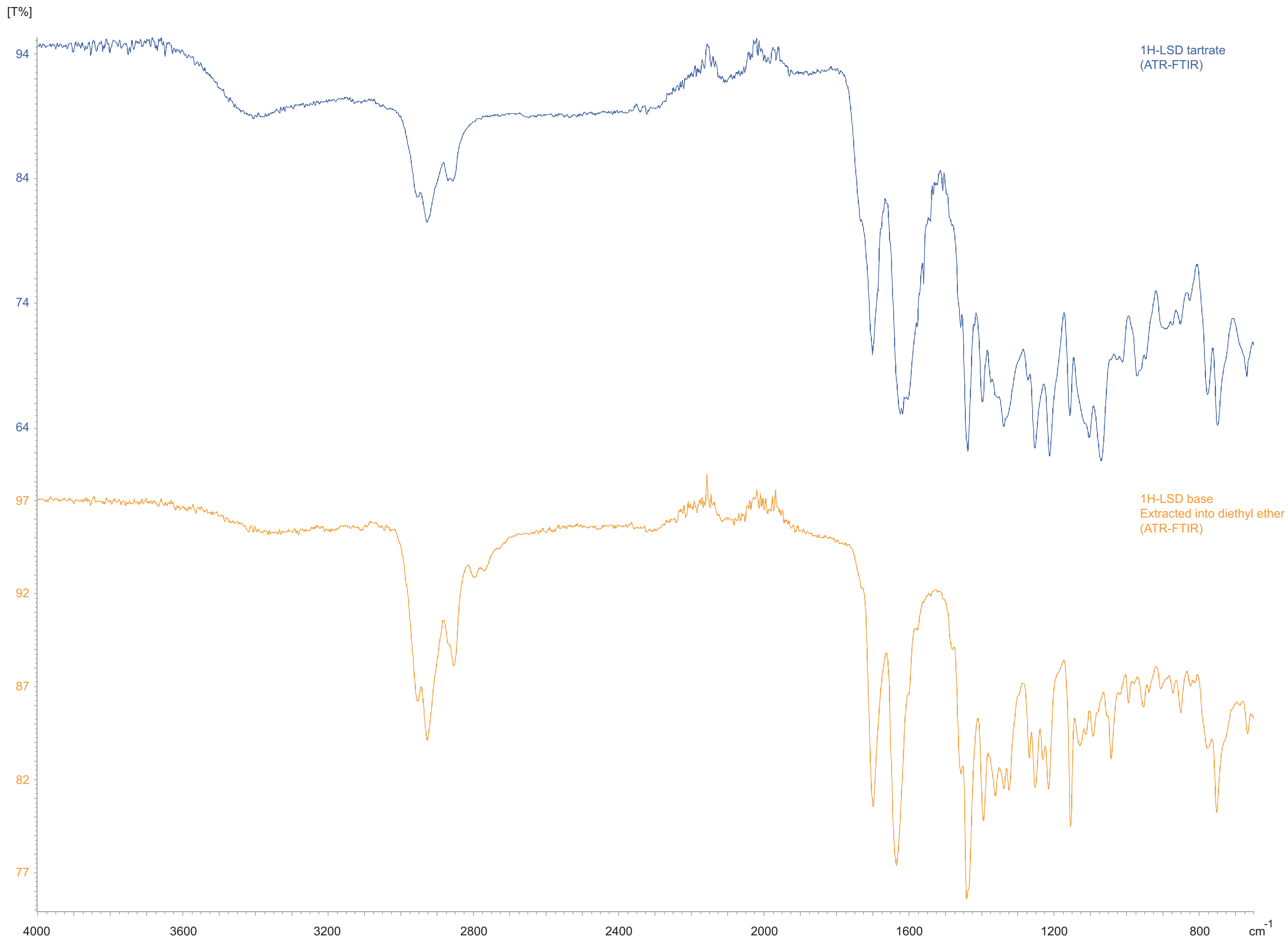
[T%]
94
84
74
64
97
92
87
82
77
1H-LSD tartrate
(ATR-FTIR)
1H-LSD base
Extracted into diethyl ether
(ATR-FTIR)
4000
3600
3200
2800
2400
2000
1600
1200
800
cm-1

An Agilent 1200 liquid chromatography system (quaternary pump G1311A, degasser G1322A, autosampler G1313A, column oven G1316A) coupled to a UV diode array detector (G1315D) was used using a Zorbax Eclipse XDB-C18 column (150 mm × 4.6 mm, 5 µm, Agilent). The column oven was set to 25°C. The mobile phases were 0.1% formic acid in water (A) and 0.1% formic acid in methanol (B). The gradient elution commenced with 95% A and ramped to 95% B in 10 min and held for 5 min, followed by a return to starting conditions at the 16 min time point. Equilibration time was set to 5 min to give a total run time of 21 min. The flow rate was 1.0 mL/min. The injection volume was 10 µL. The diode array detection window was set at 210–450 nm (2 nm steps).

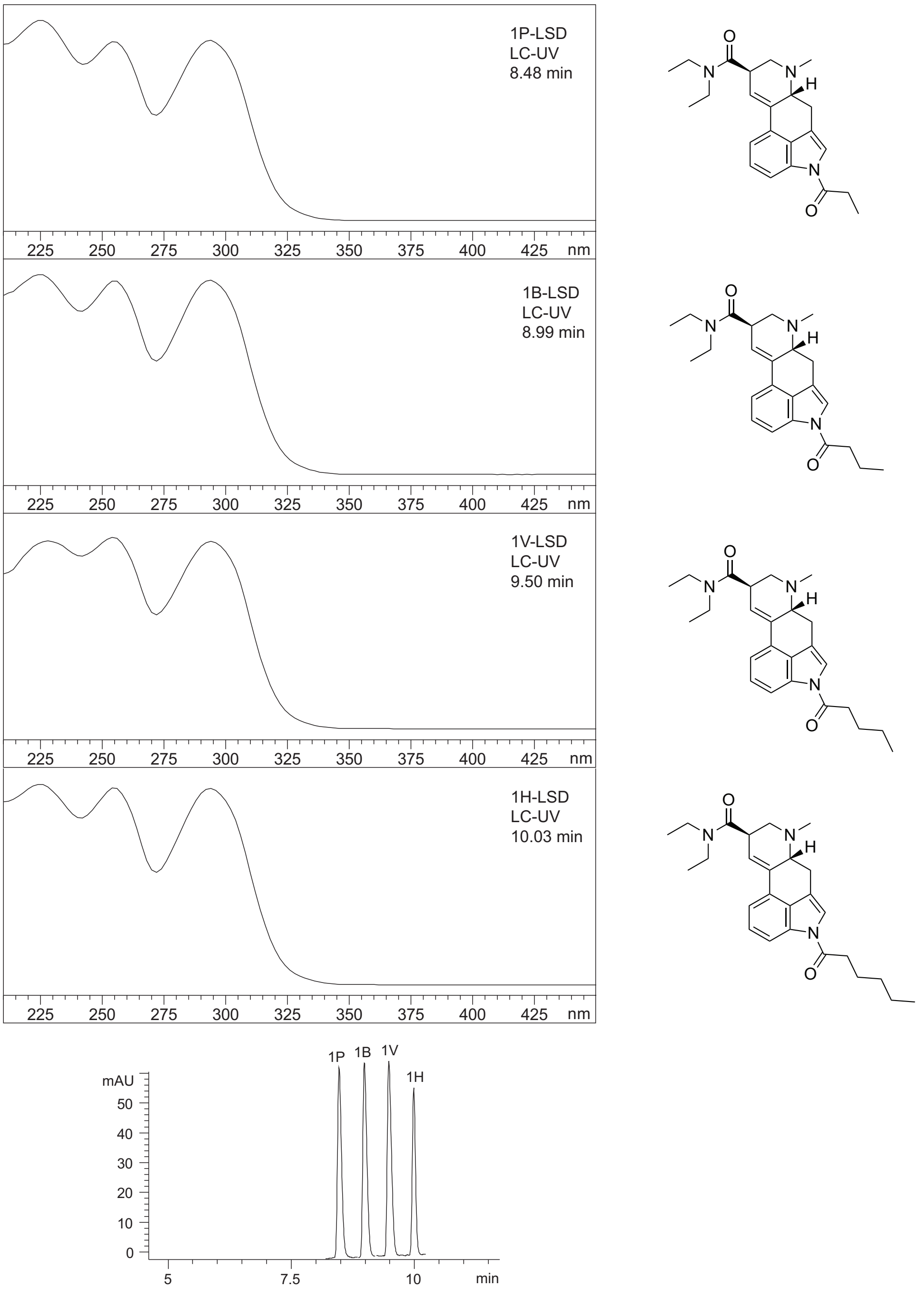

1H-LSD tartrate
1H NMR (600 MHz)
DMSO-d_6

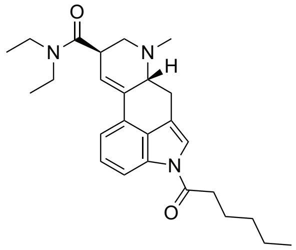

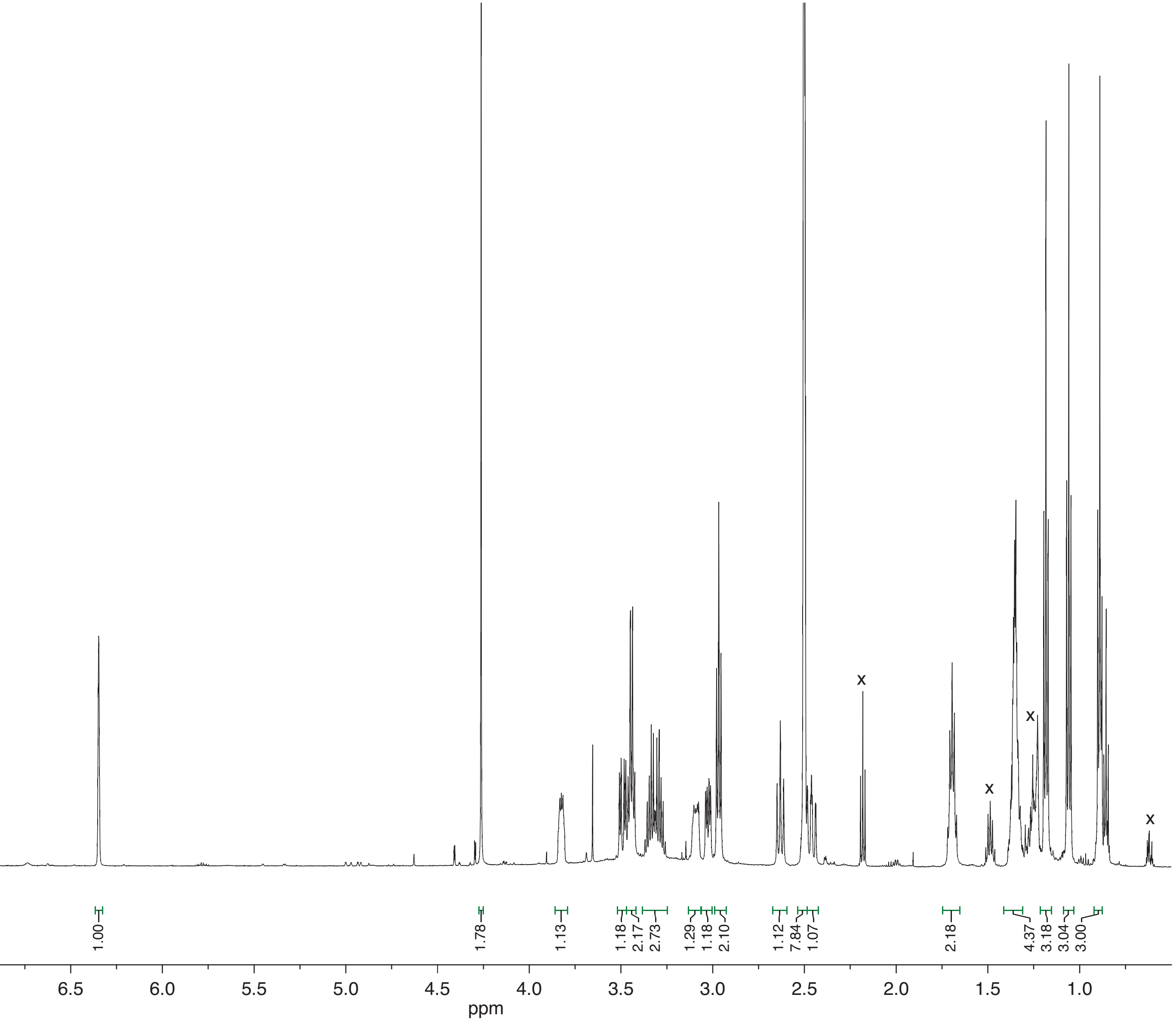

X: Hexanoic acid

1H-LSD tartrate
1H NMR (600 MHz)
DMSO-d_6

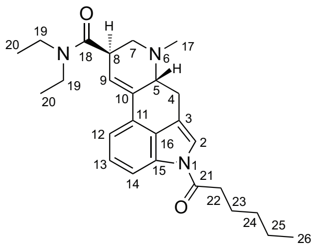

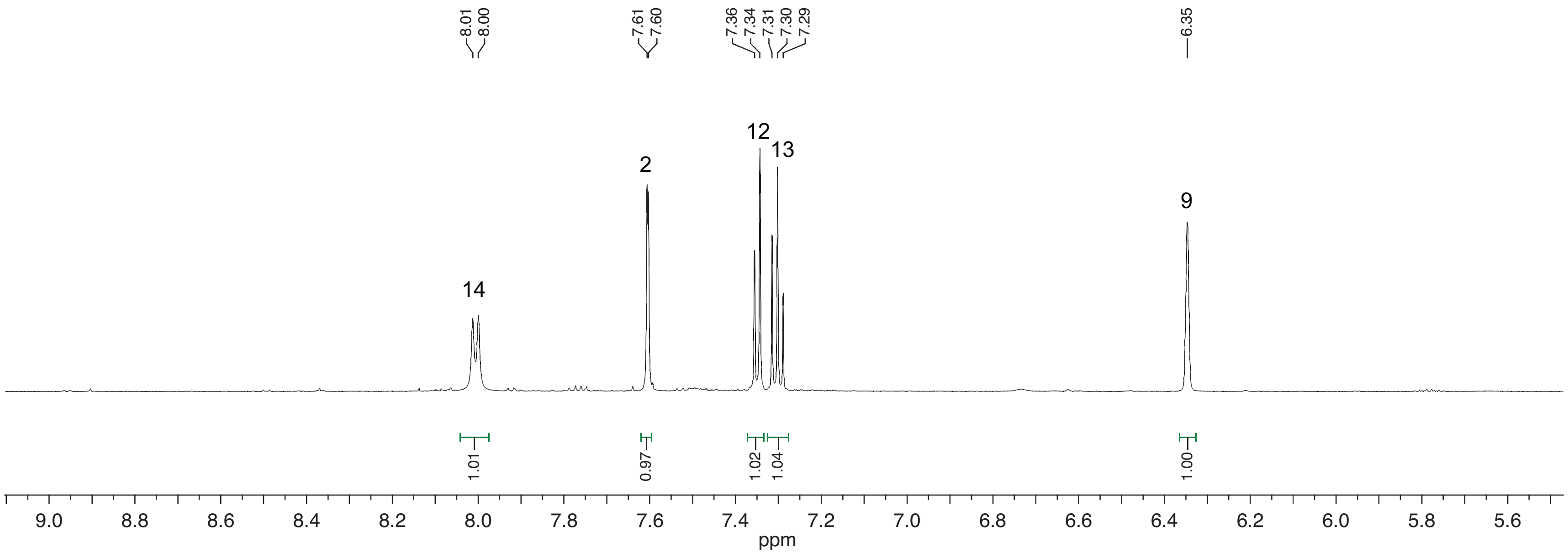

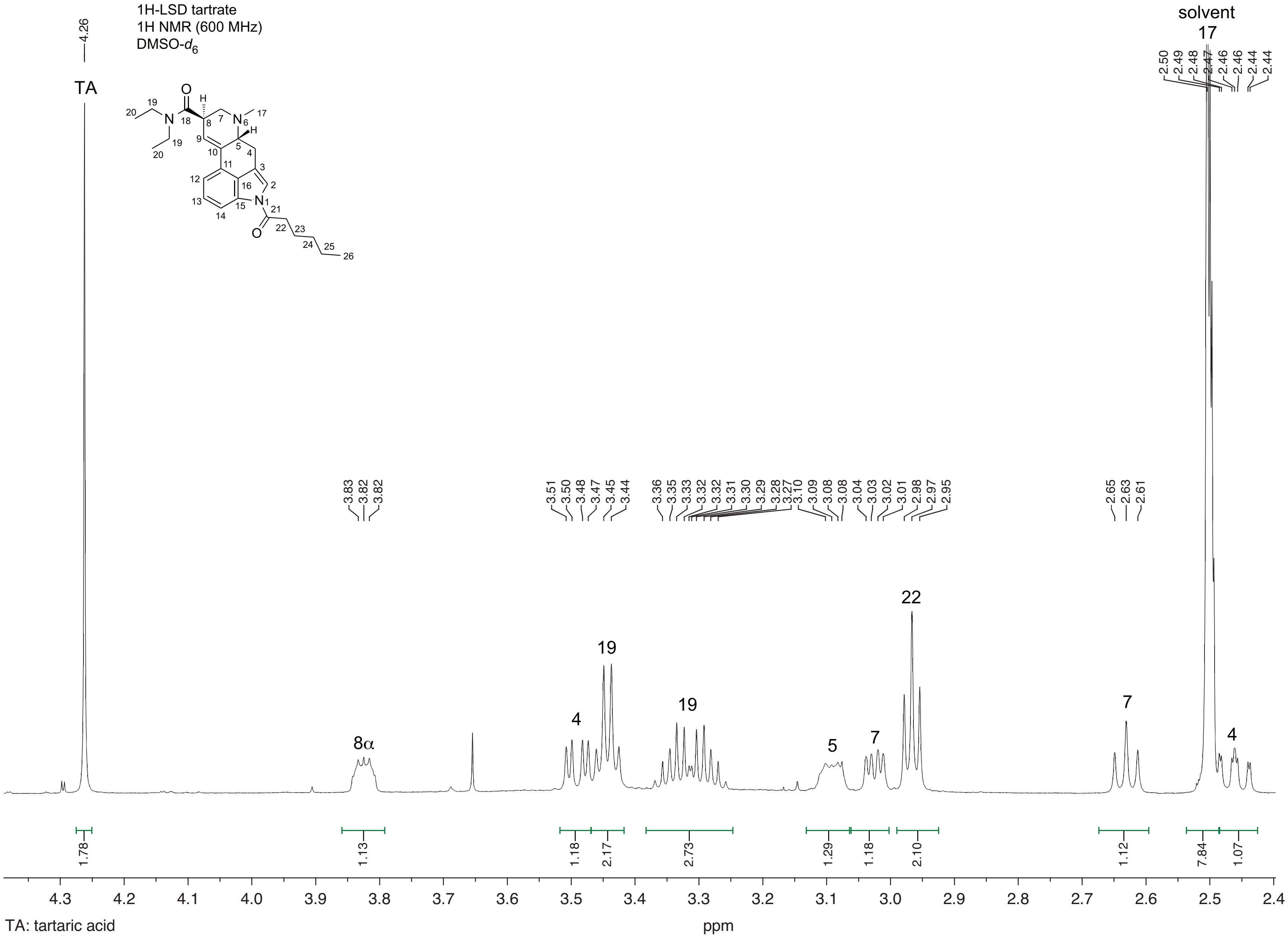
1H-LSD tartrate
1H NMR (600 MHz)
DMSO-d_6
TA
solvent
17
8α
4
19
19
5
7
22
7
4
ppm
TA: tartaric acid

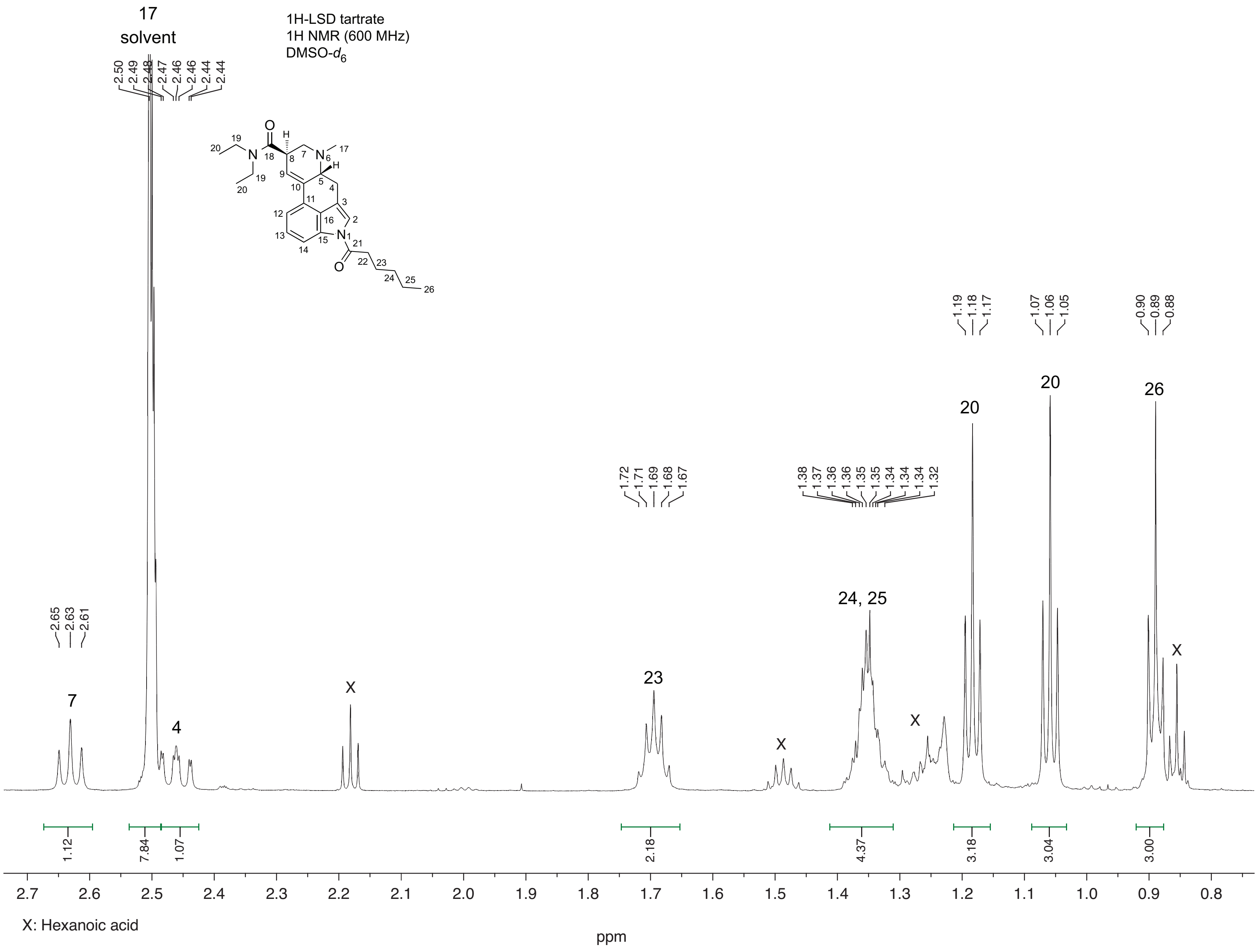
1H-LSD tartrate
1H NMR (600 MHz)
DMSO-d_6
17
solvent
2.50 2.49 2.48 2.47 2.46 2.46 2.44 2.44
2.65 2.63 2.61
7
4
X
1.72 1.71 1.69 1.68 1.67
23
1.38 1.37 1.36 1.36 1.35 1.35 1.34 1.34 1.34 1.32
24, 25
X
X
1.19 1.18 1.17
20
1.07 1.06 1.05
20
0.90 0.89 0.88
26
X
1.12
7.84
1.07
2.18
4.37
3.18
3.04
3.00
2.7 2.6 2.5 2.4 2.3 2.2 2.1 2.0 1.9 1.8 1.7 1.6 1.5 1.4 1.3 1.2 1.1 1.0 0.9 0.8
ppm
X: Hexanoic acid

1H-LSD tartrate
COSY NMR (600 MHz)
DMSO-d_6

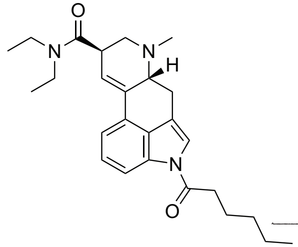

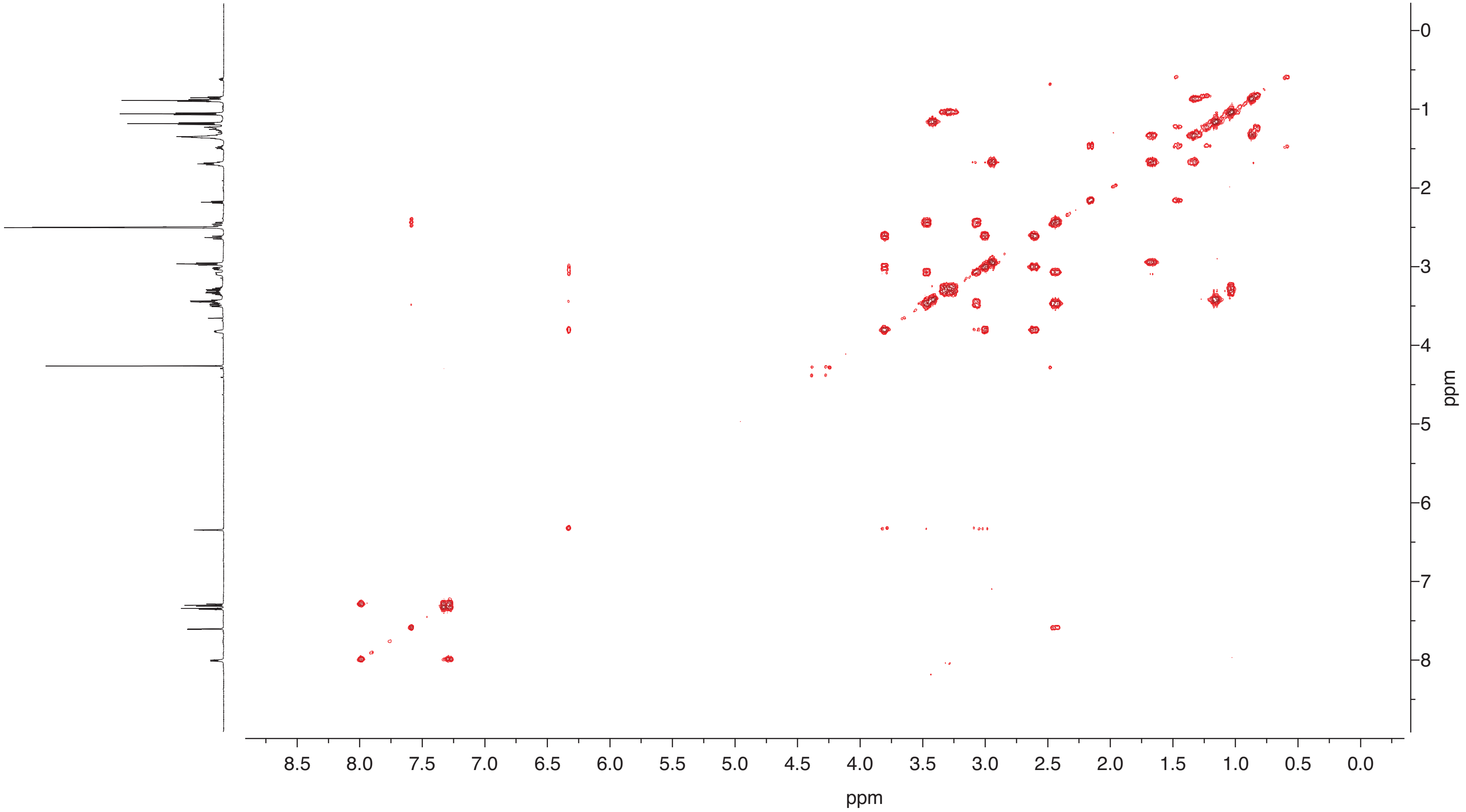

1H-LSD tartrate
COSY (600 MHz)
DMSO-d_6

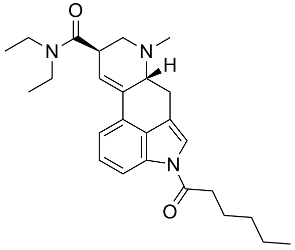

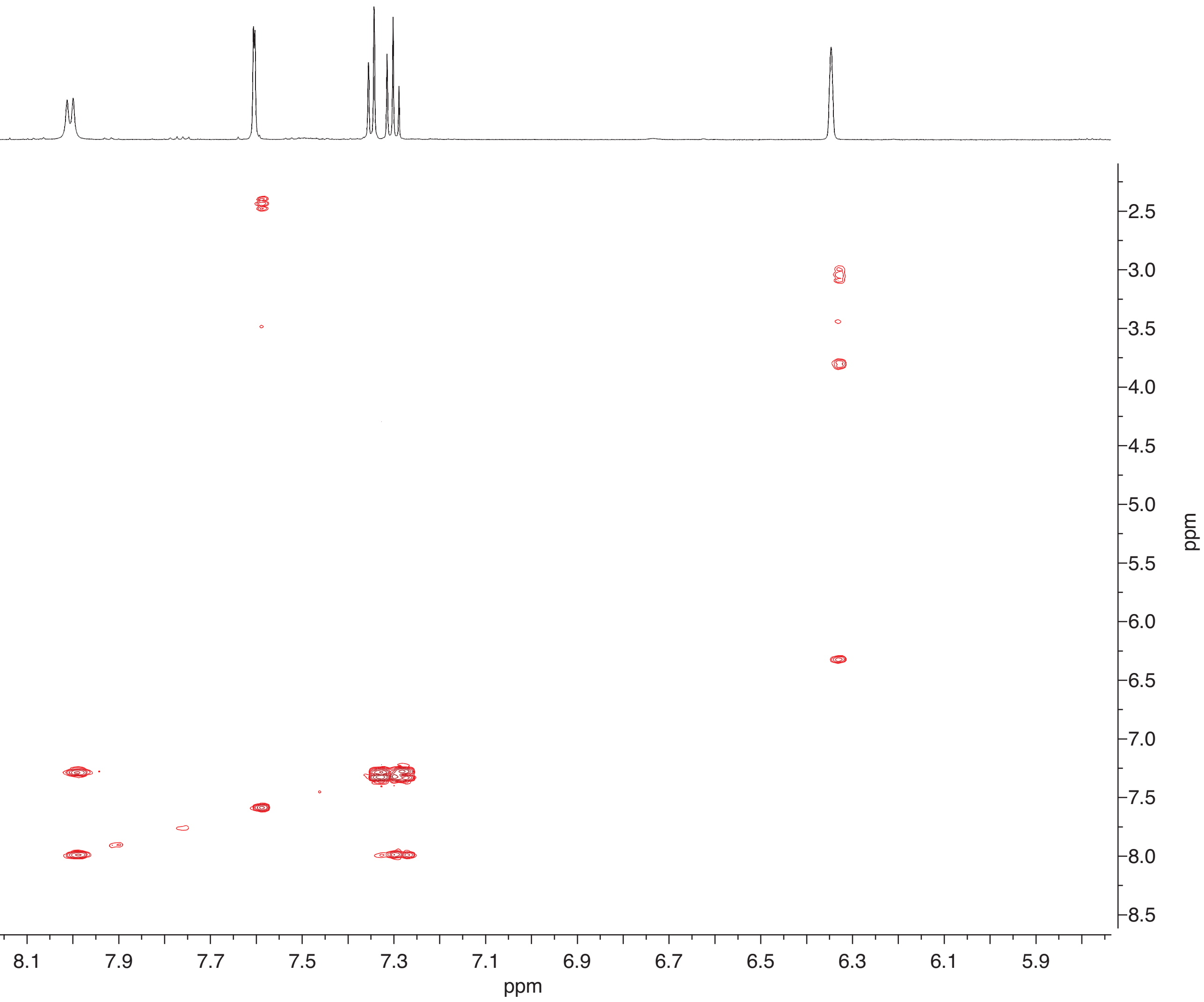

1H-LSD tartrate
COSY (600 MHz)
DMSO-d_6

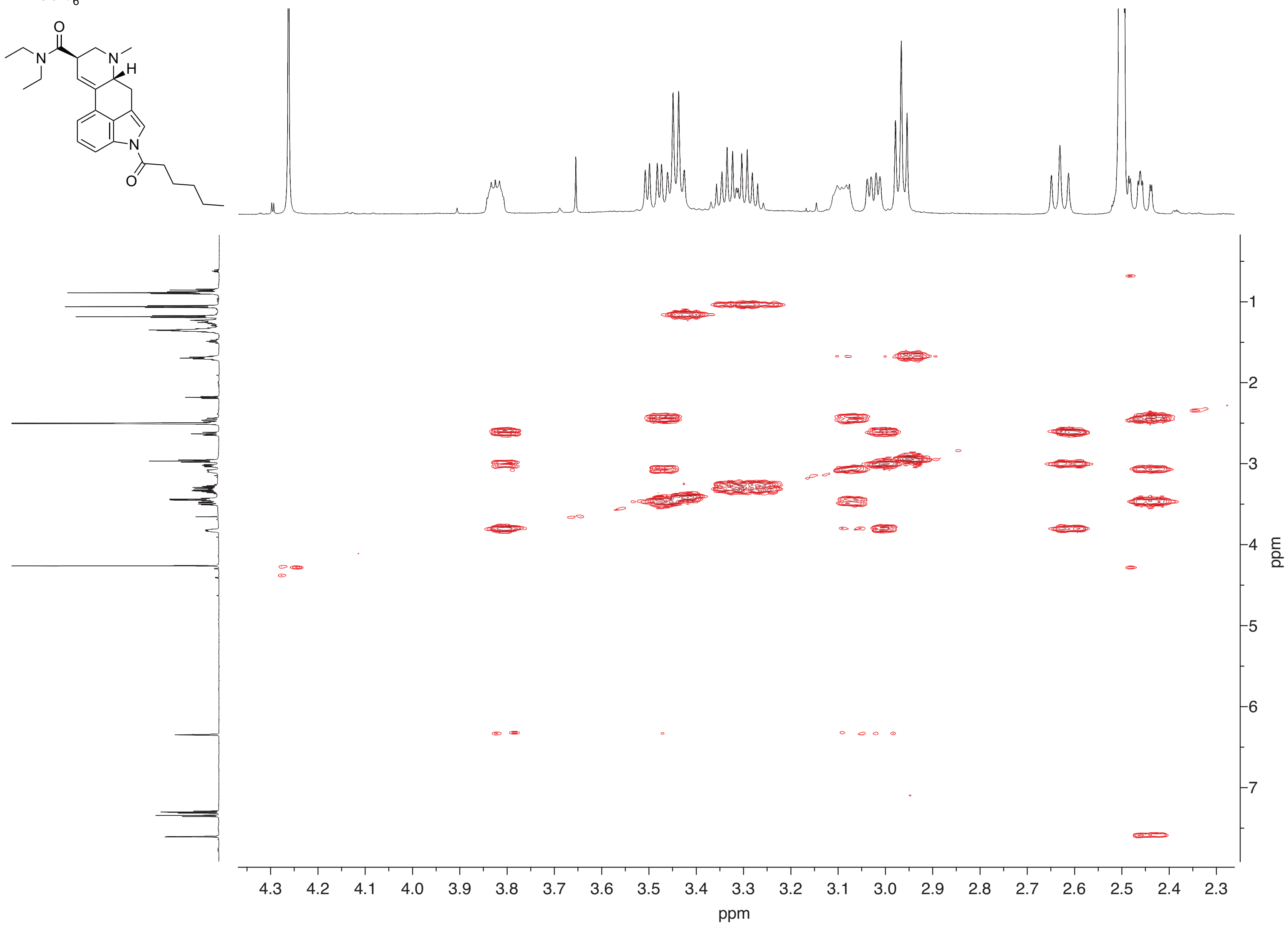

1H-LSD tartrate
COSY (600 MHz)
DMSO-d_6

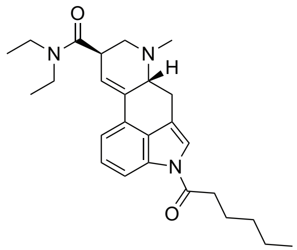

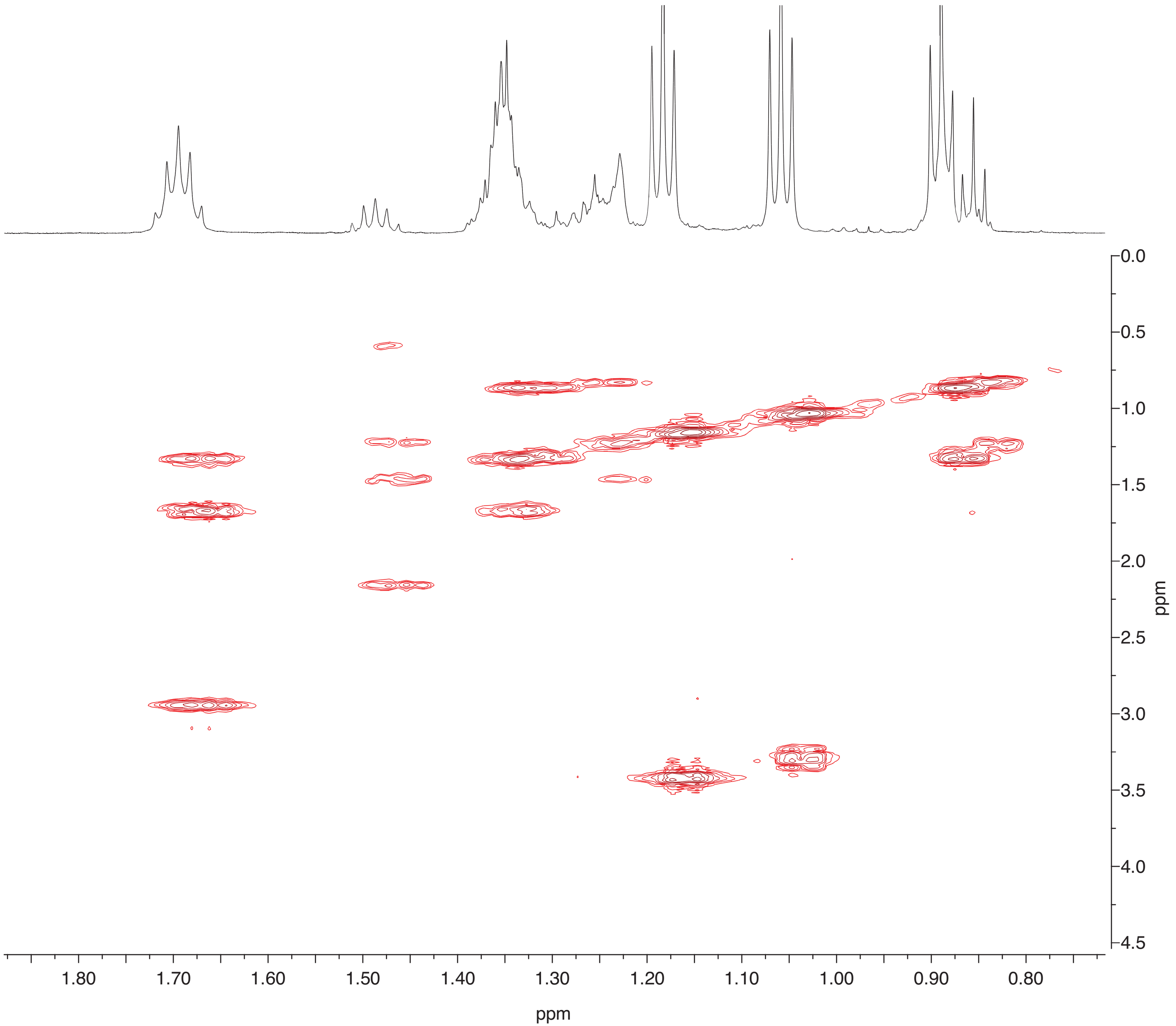

1H-LSD tartrate
13C NMR (175 MHz)
DMSO-d_6

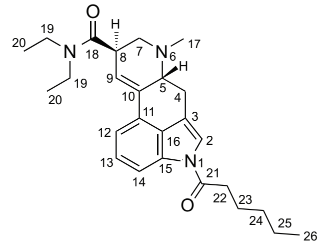

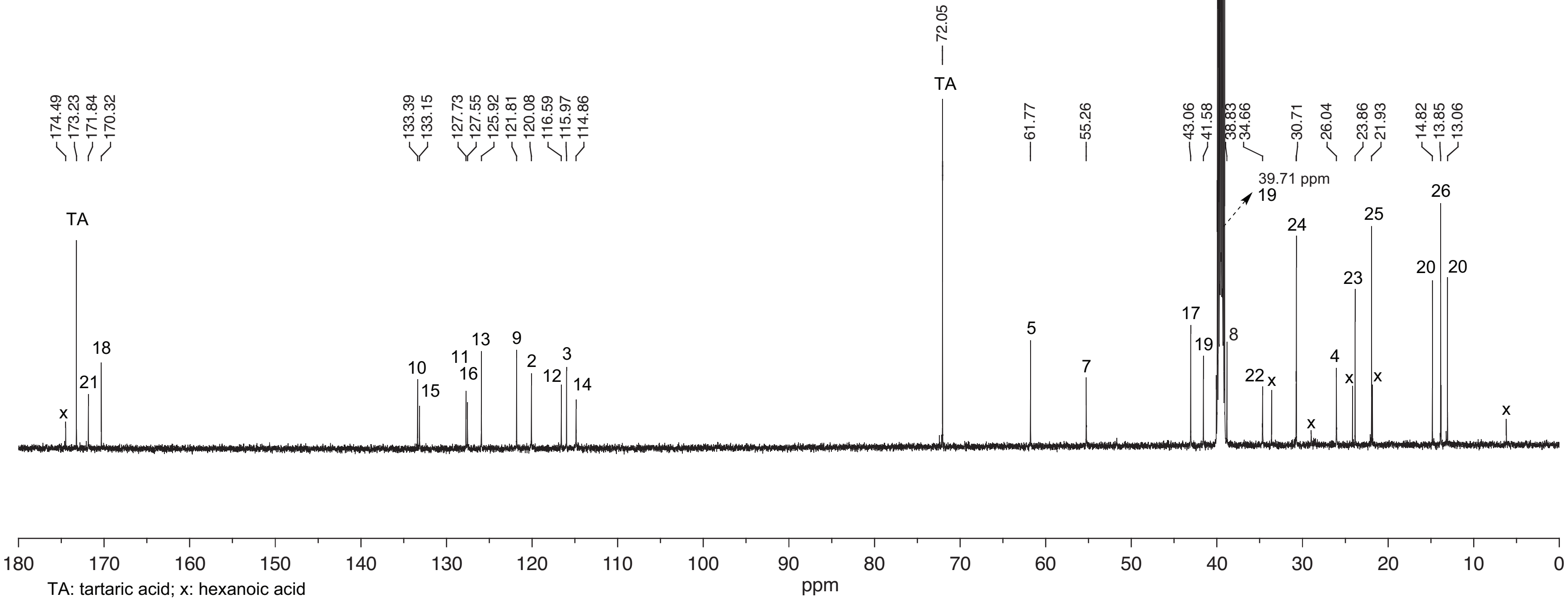

TA: tartaric acid; x: hexanoic acid

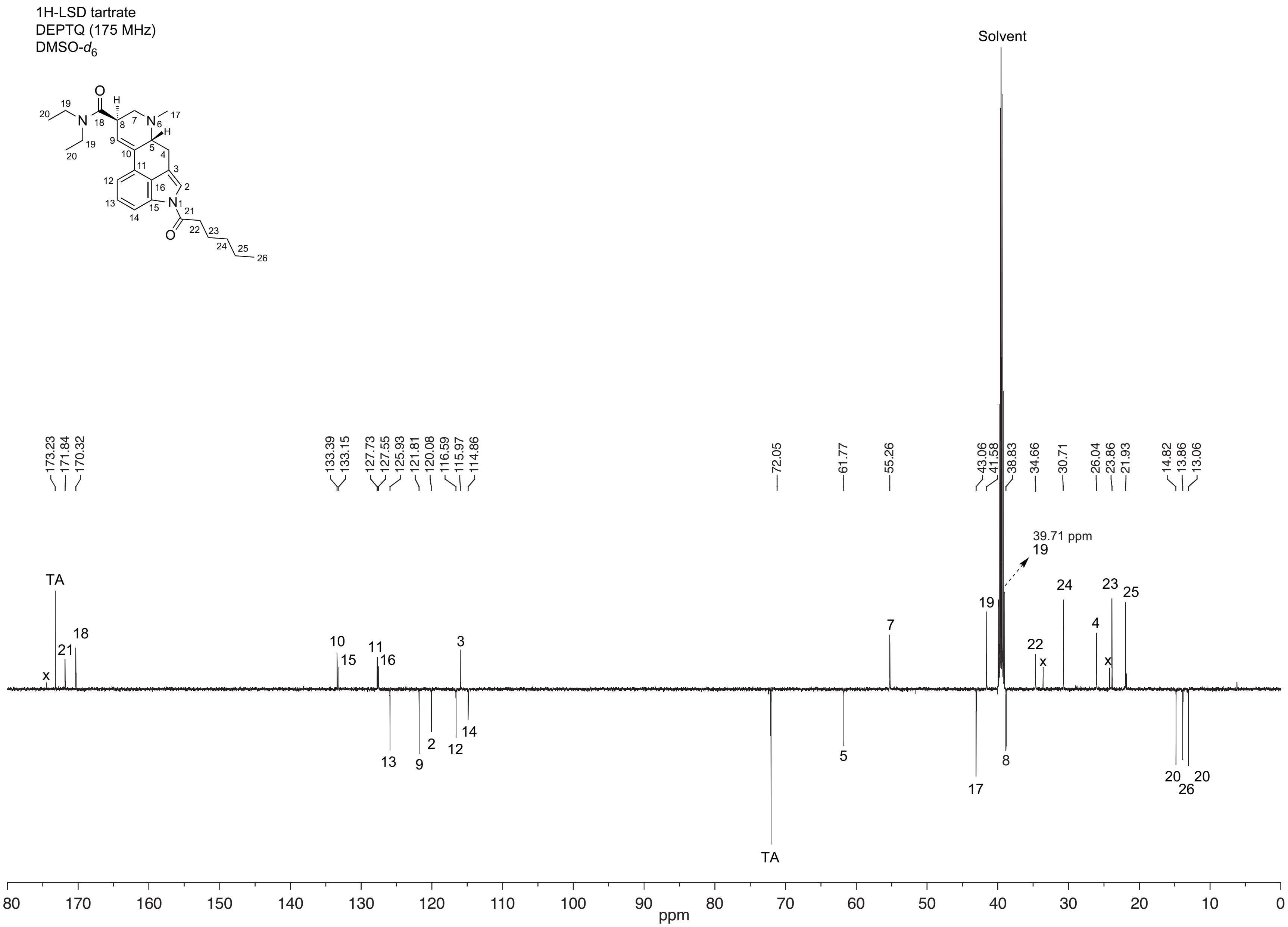

TA: tartaric acid; x: hexanoic acid

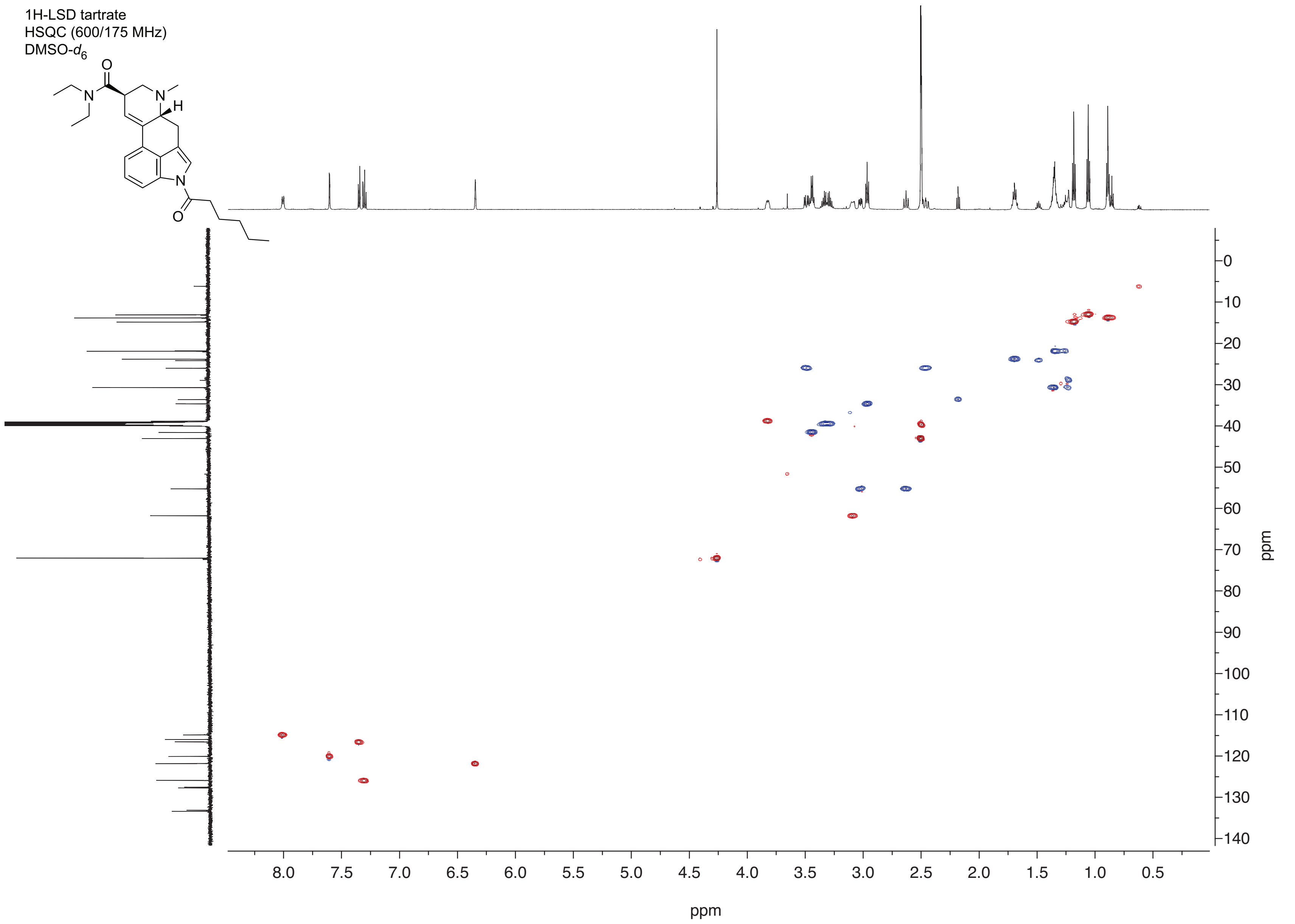
1H-LSD tartrate
HSQC (600/175 MHz)
DMSO-d_6
ppm
ppm

1H-LSD tartrate
HSQC (600/175 MHz)
DMSO-d_6

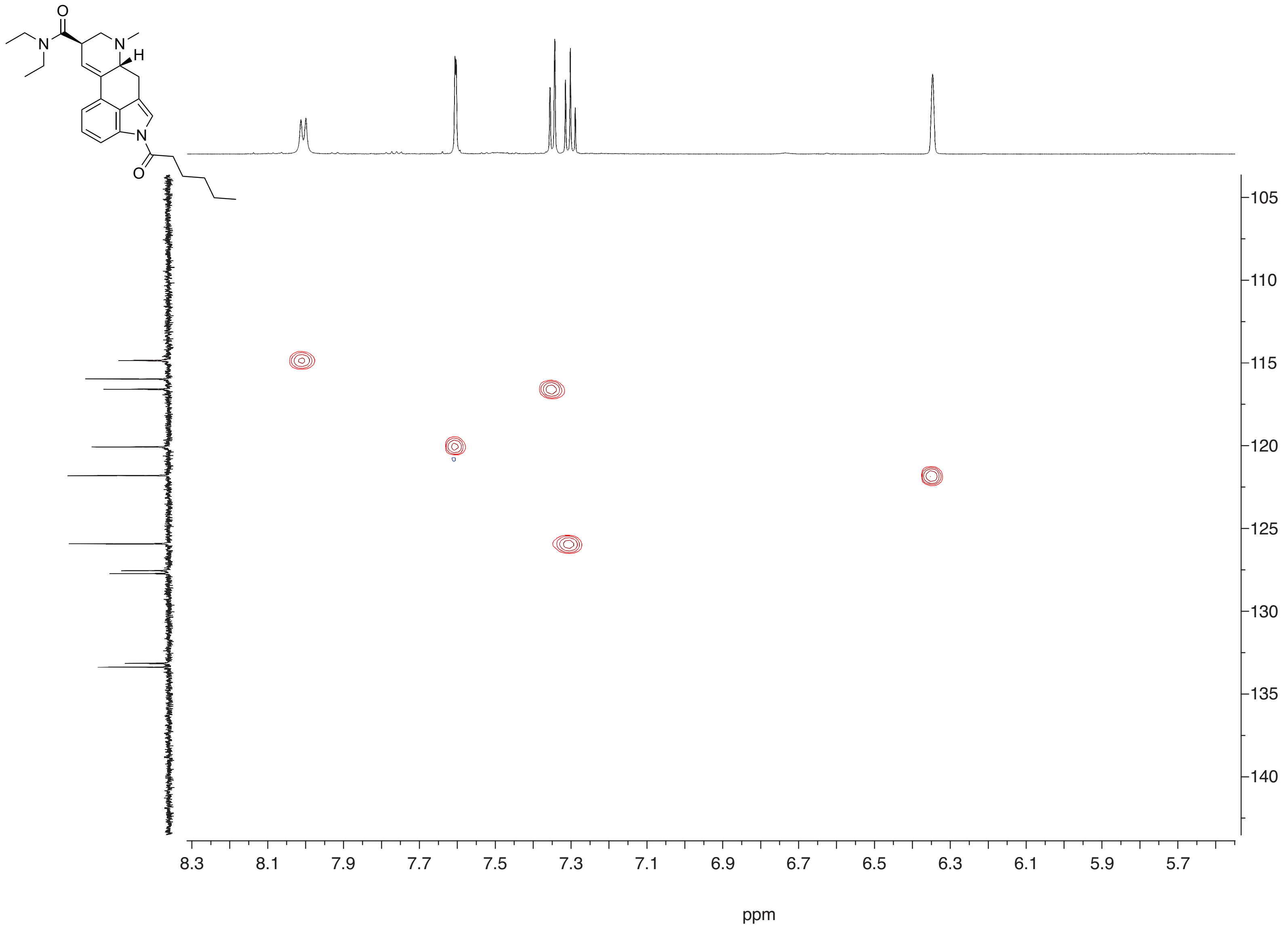

1H-LSD tartrate
HSQC (600/175 MHz)
DMSO-d_6

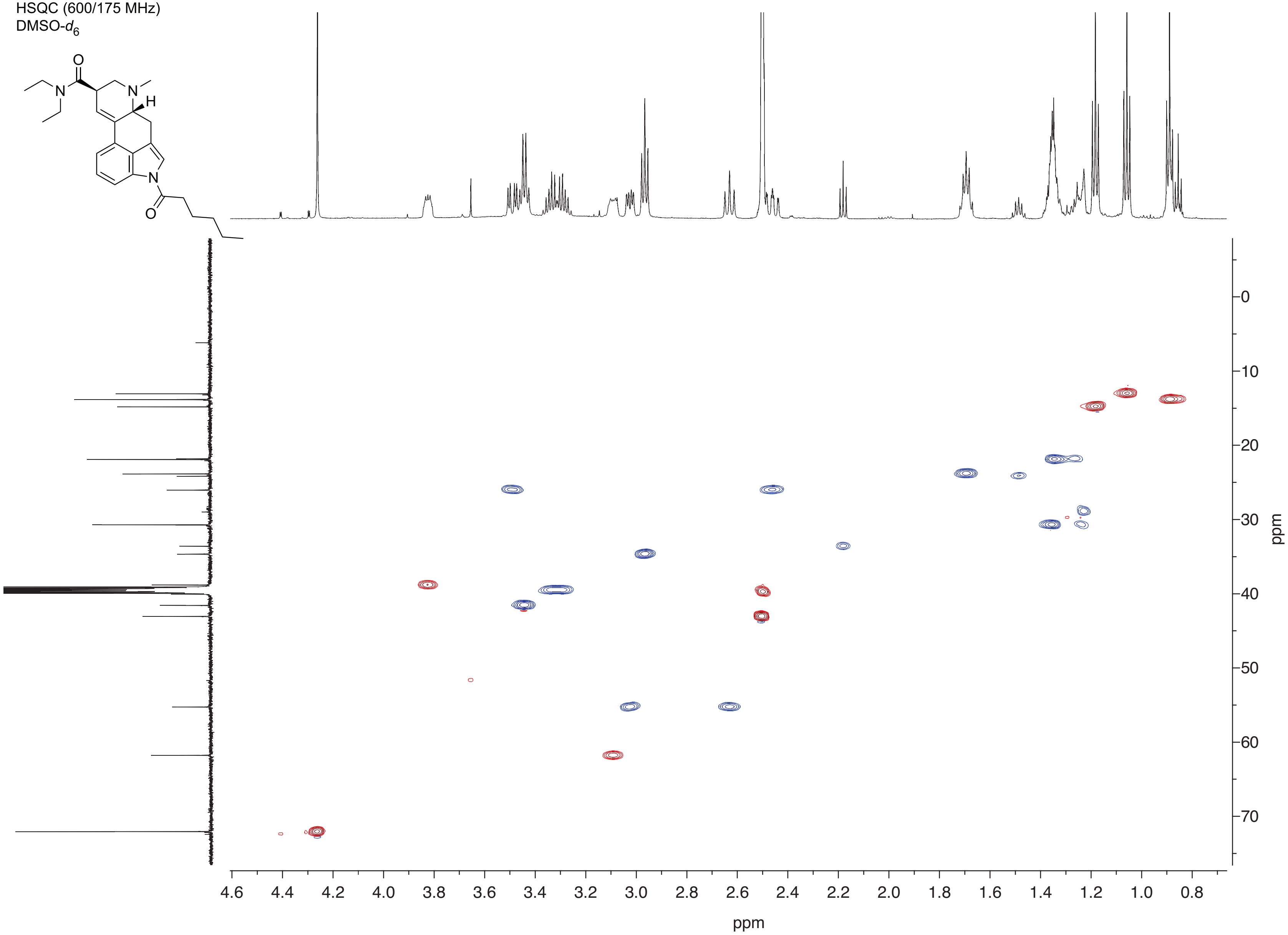

1H-LSD tartrate
HSQC (600/175 MHz)
DMSO-d_6

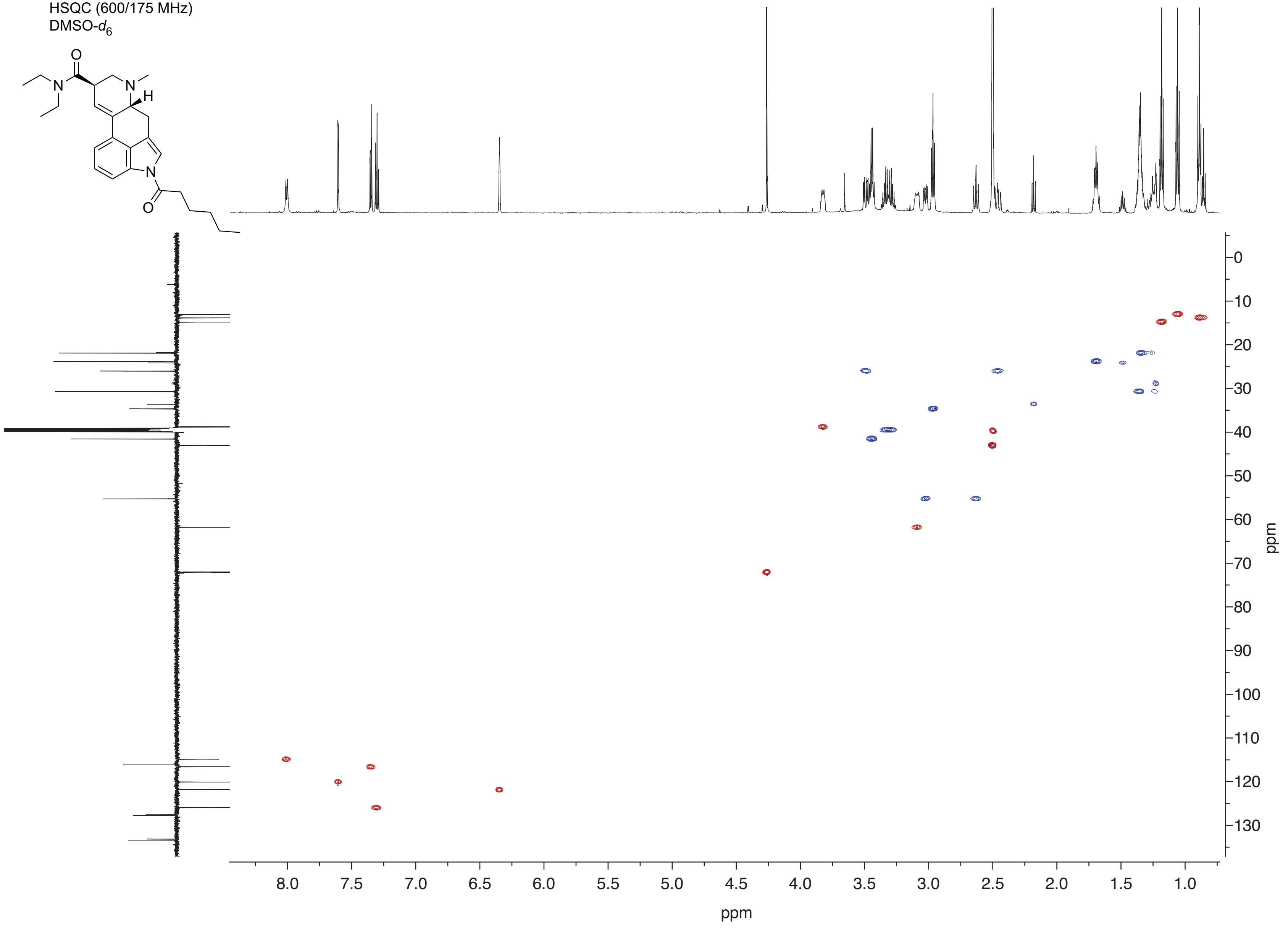

1H-LSD tartrate
HSQC (600/175 MHz)
DMSO-d_6

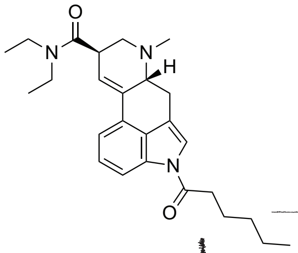

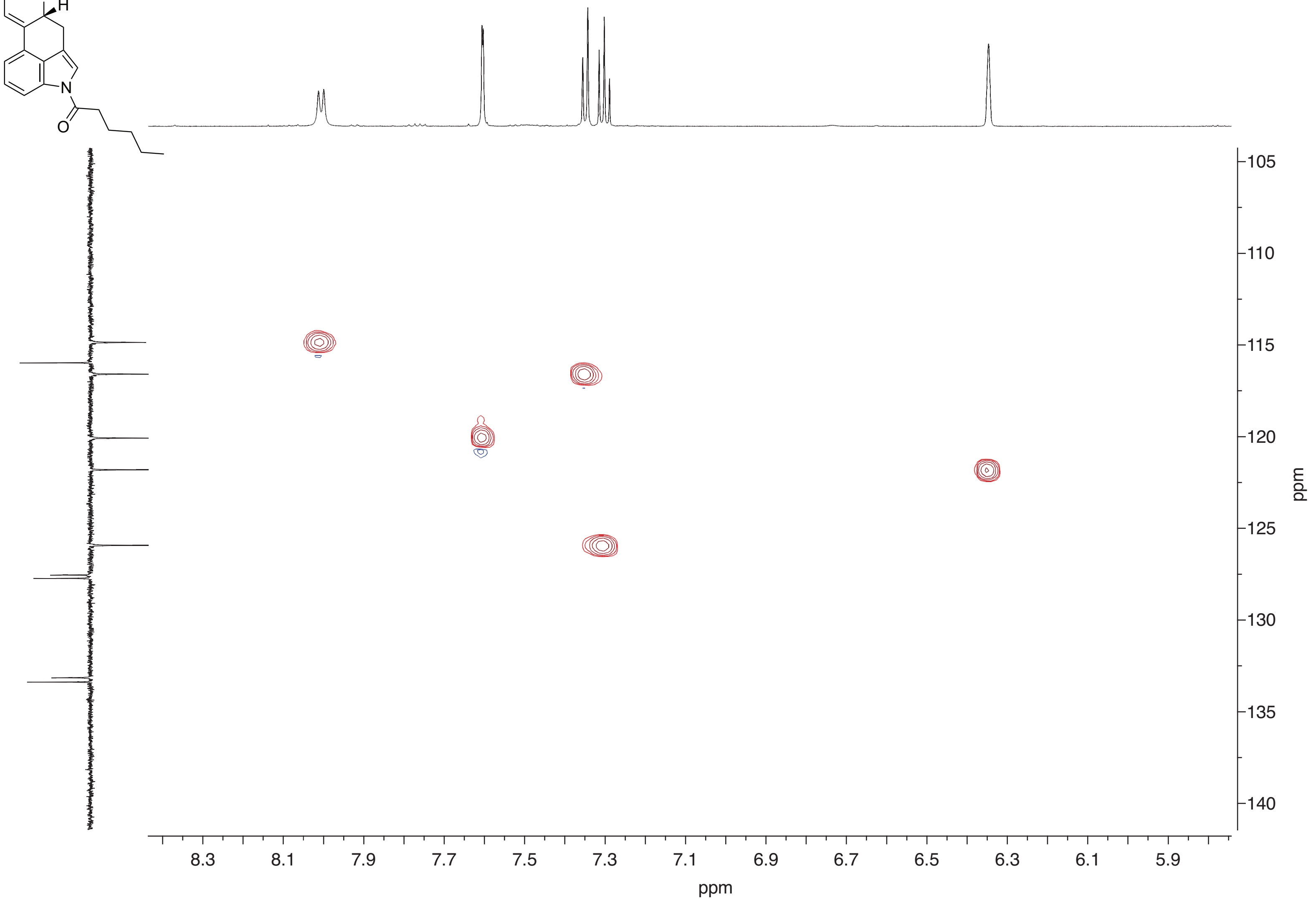

1H-LSD tartrate
HSQC (600/175 MHz)
DMSO-d_6

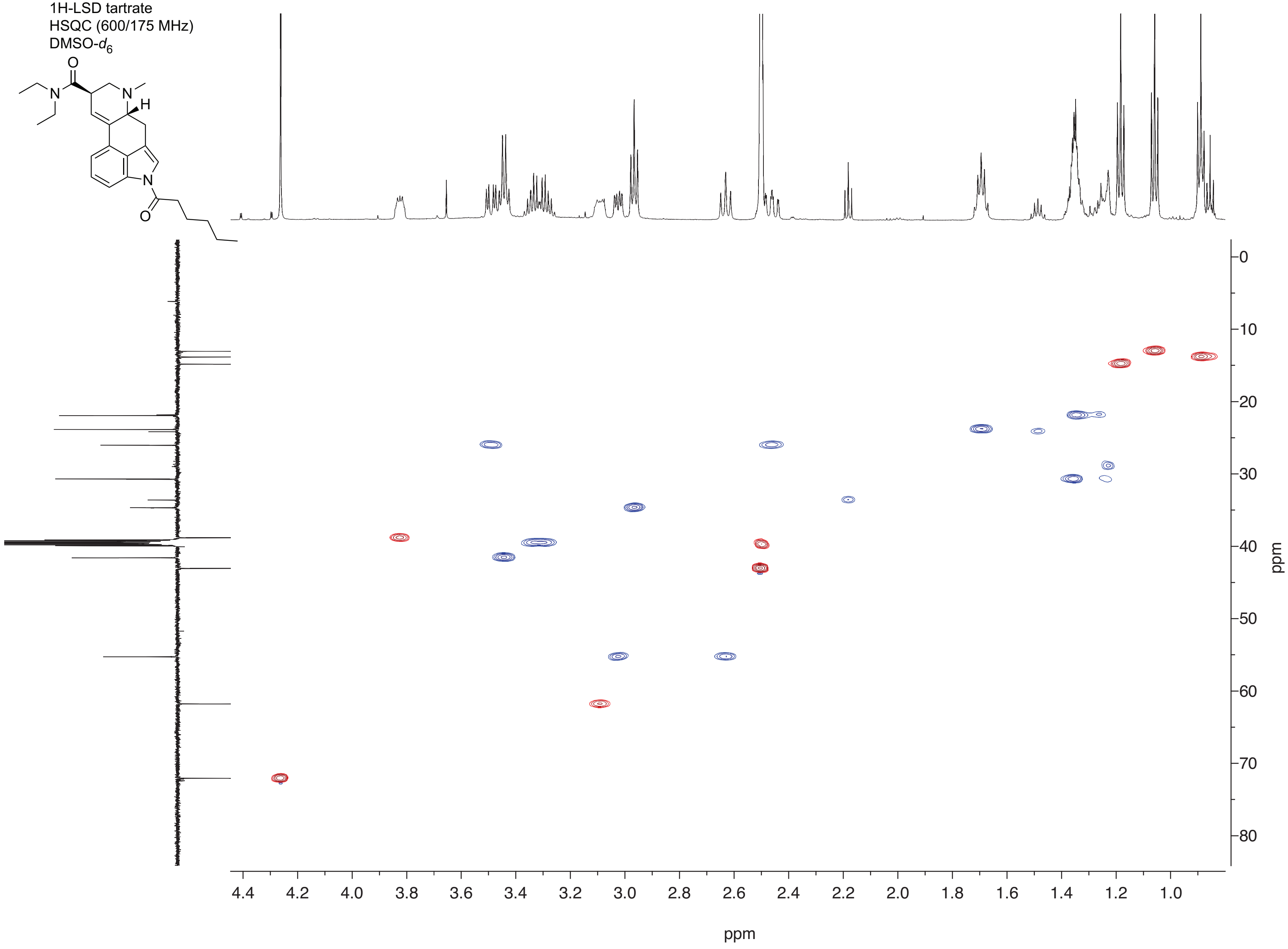

1H-LSD tartrate
HMBC (600/175 MHz)
DMSO-d_6

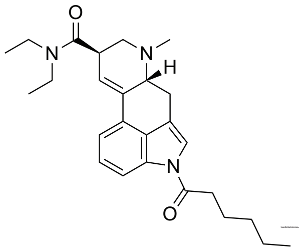

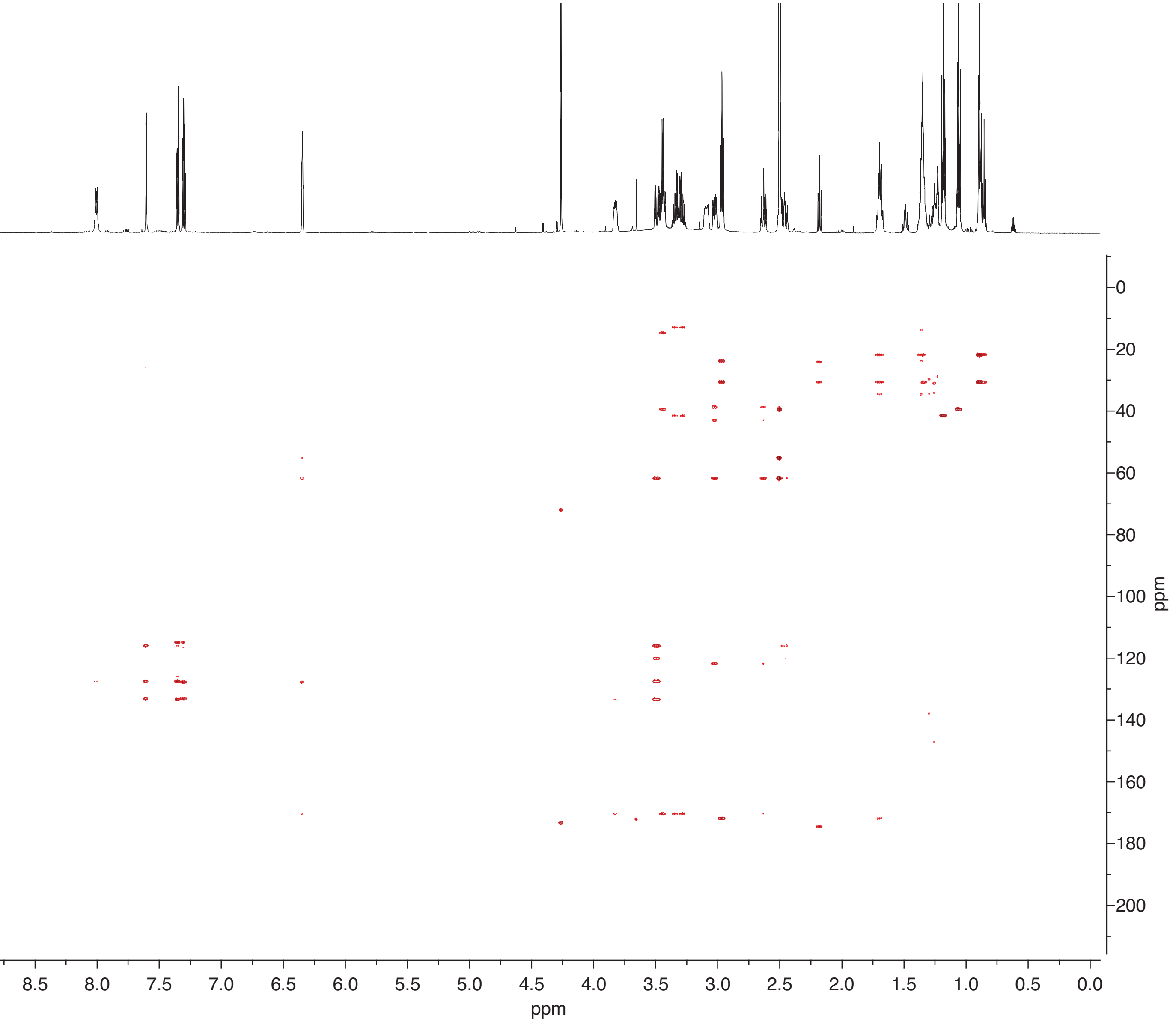

1H-LSD tartrate
HMBC (600/175 MHz)
DMSO-d_6

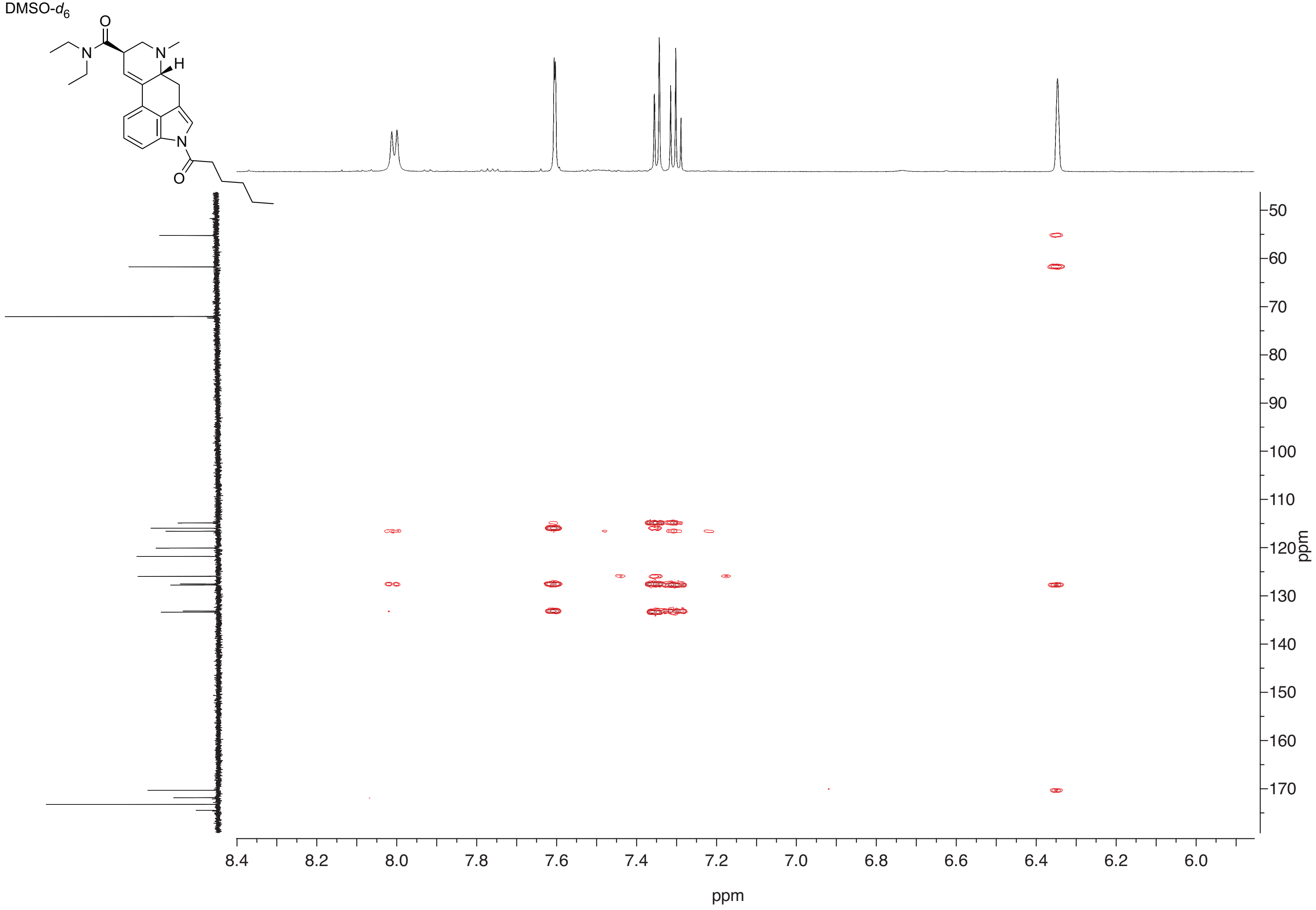

1H-LSD tartrate
HMBC (600/175 MHz)
DMSO-d_6

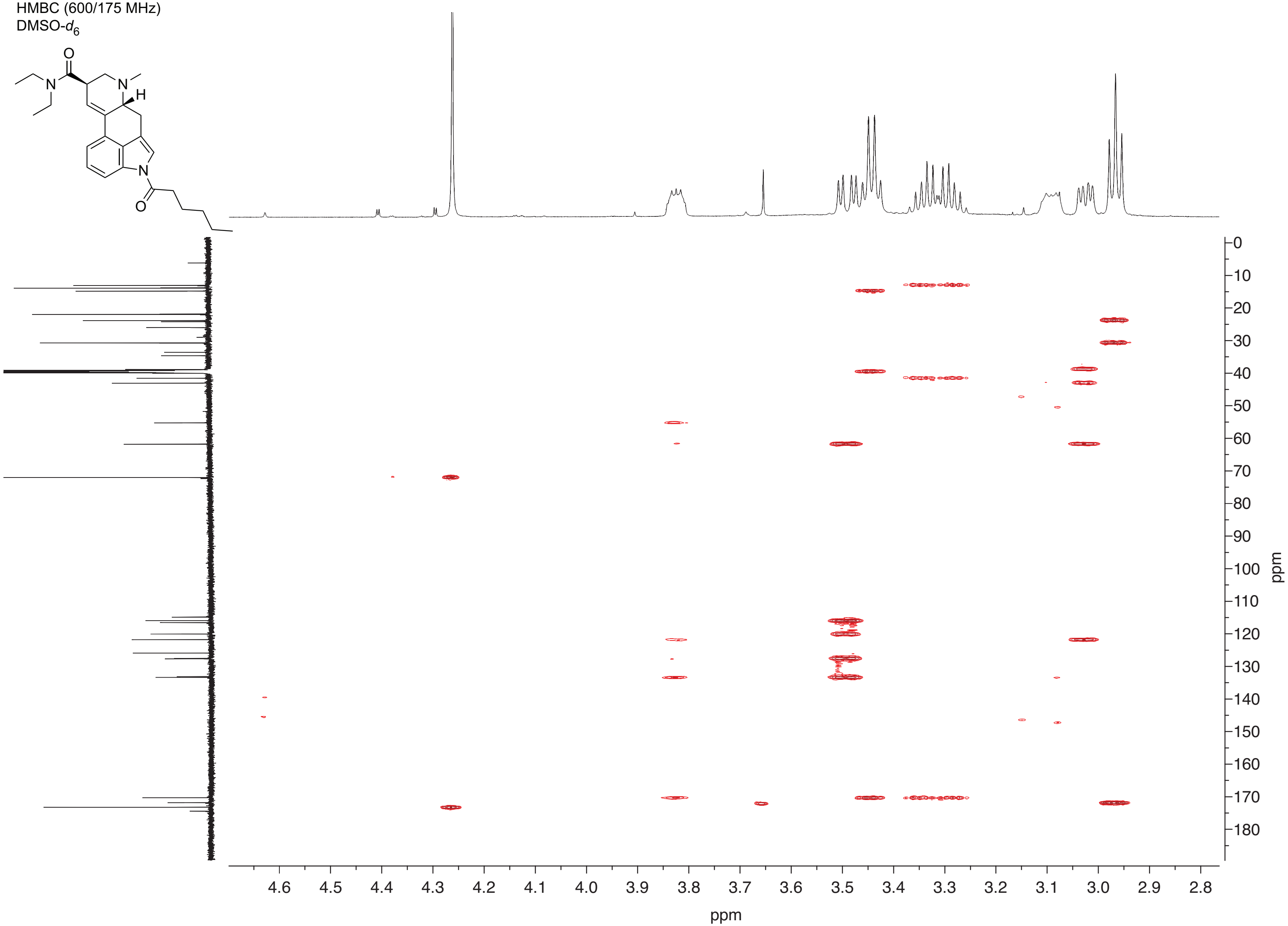

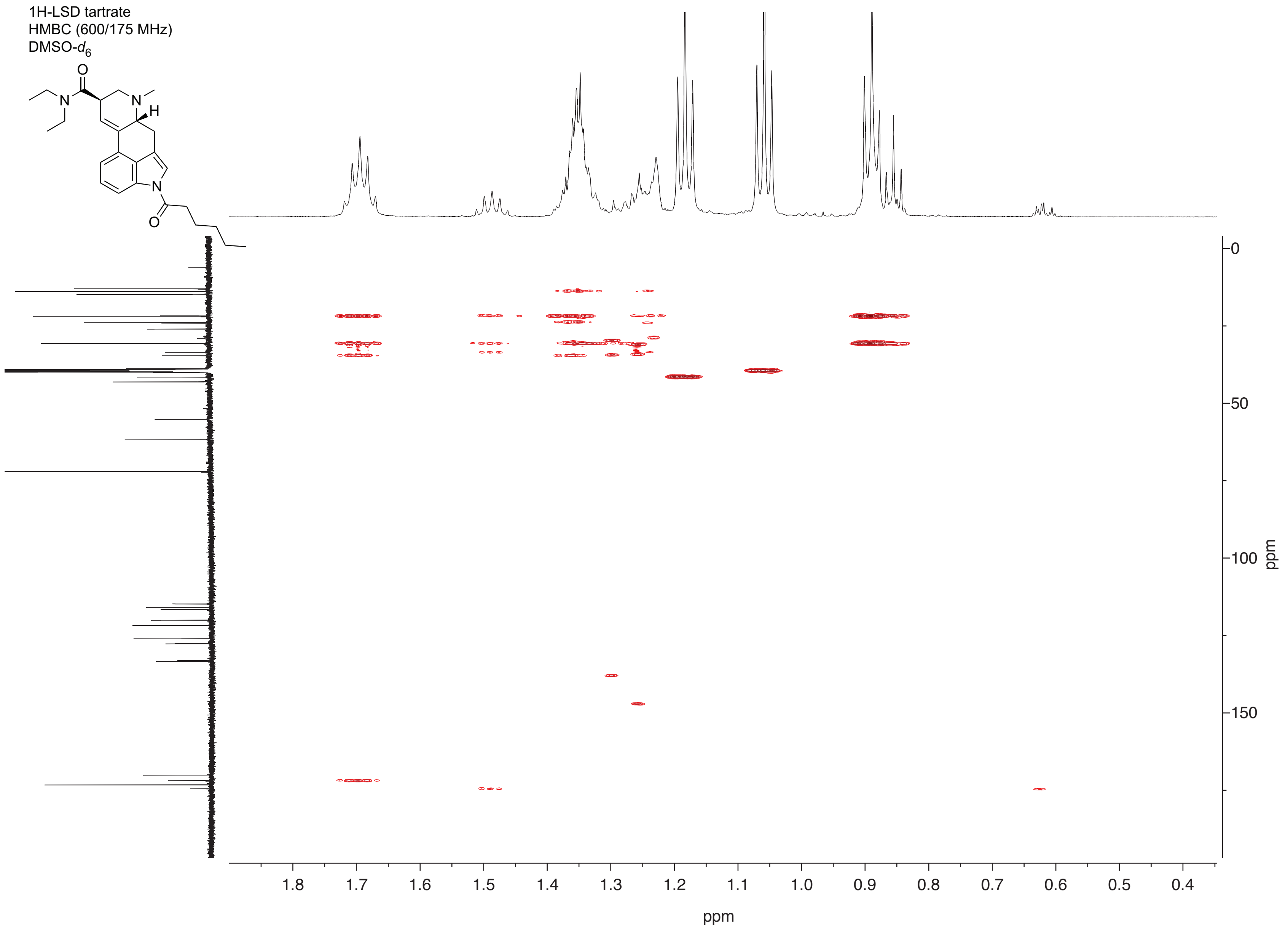
1H-LSD tartrate
HMBC (600/175 MHz)
DMSO-d_6
O
N
N
H
N
O
0
50
100
150
ppm
1.8
1.7
1.6
1.5
1.4
1.3
1.2
1.1
1.0
0.9
0.8
0.7
0.6
0.5
0.4
ppm

1H-LSD tartrate
HMBC (600/175 MHz)
DMSO-d_6

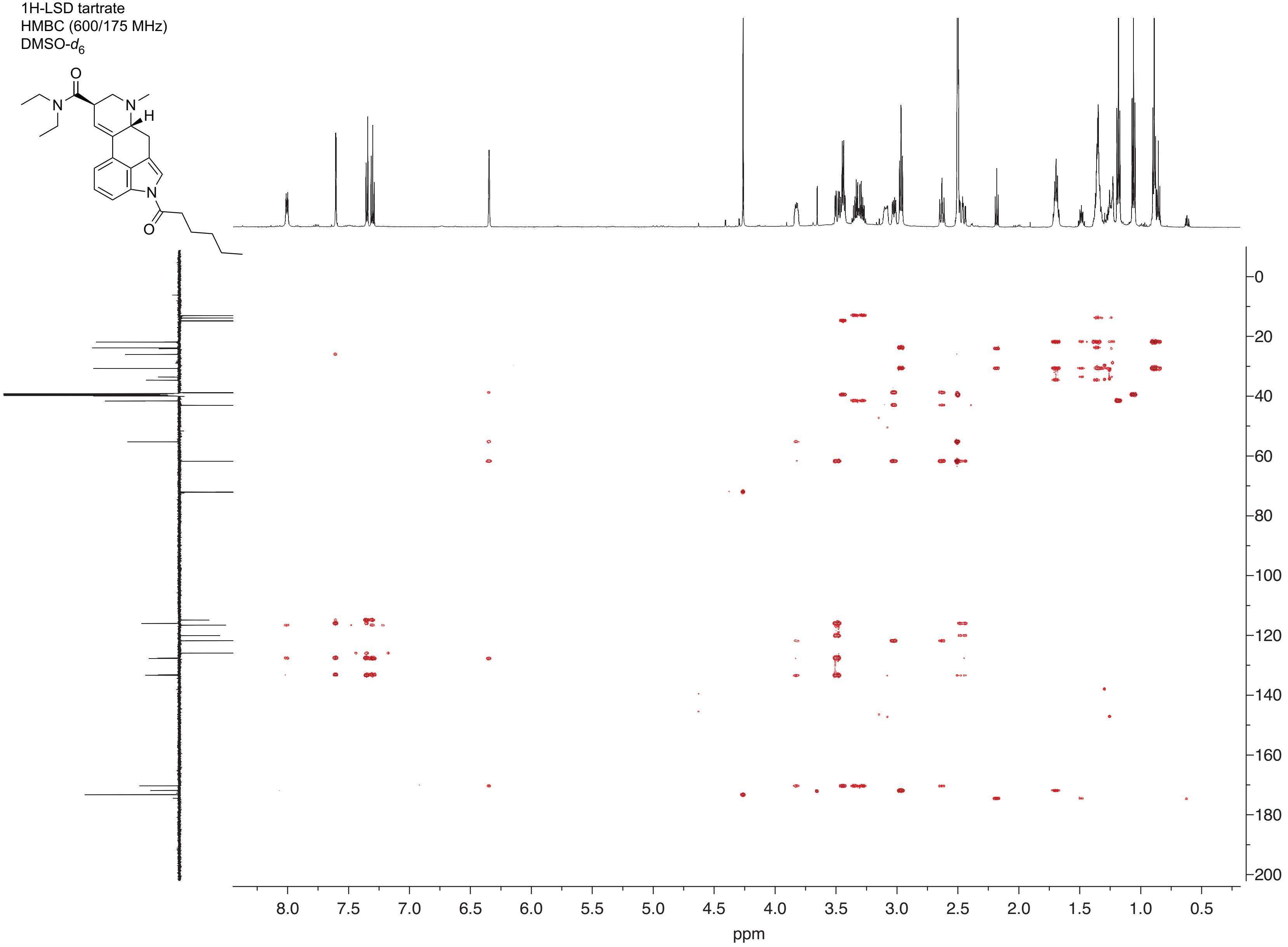

1H-LSD tartrate
HMBC (600/175 MHz)
DMSO-d_6

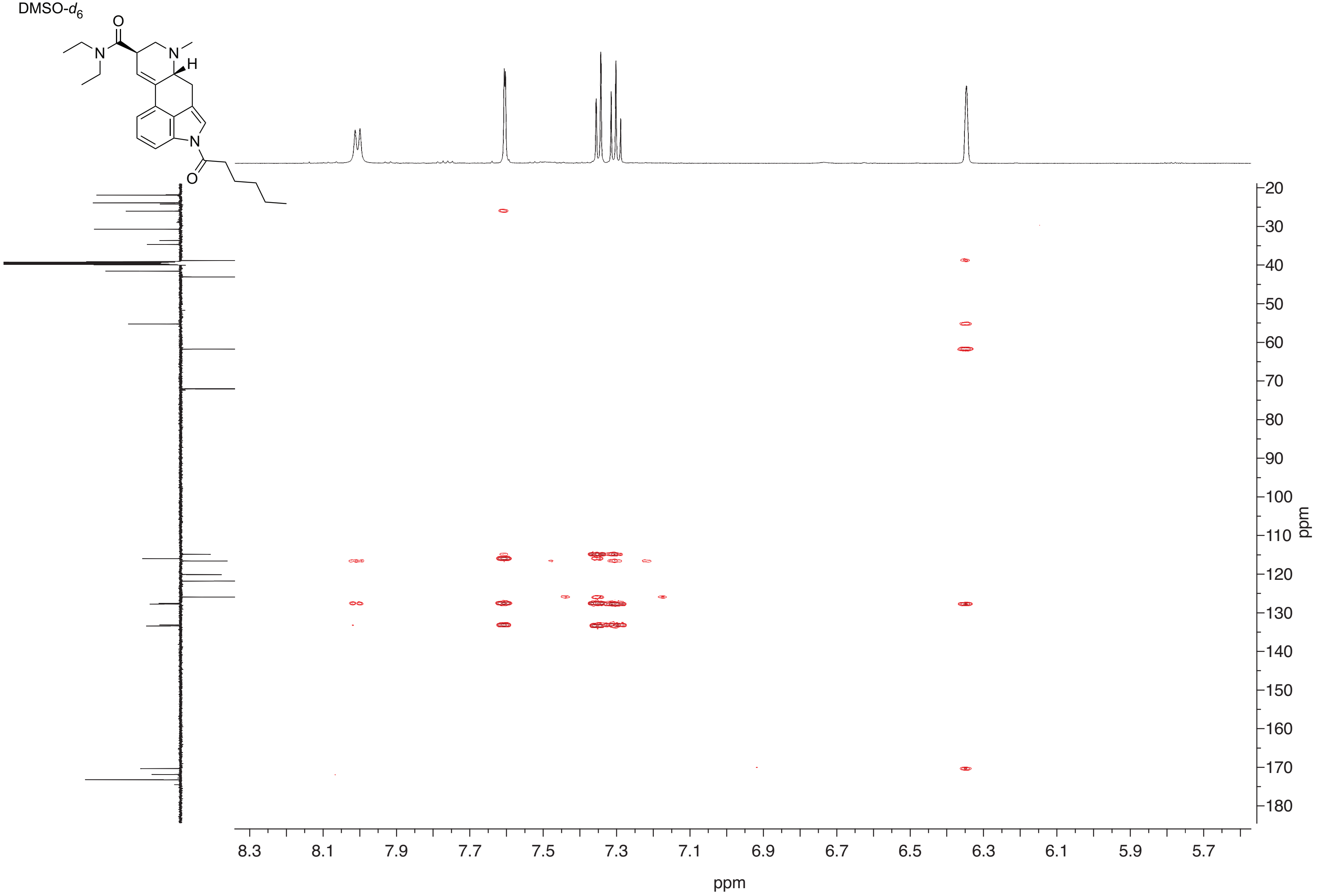

1H-LSD tartrate
HMBC (600/175 MHz)
DMSO-d_6

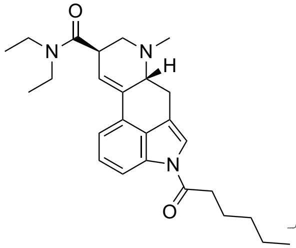

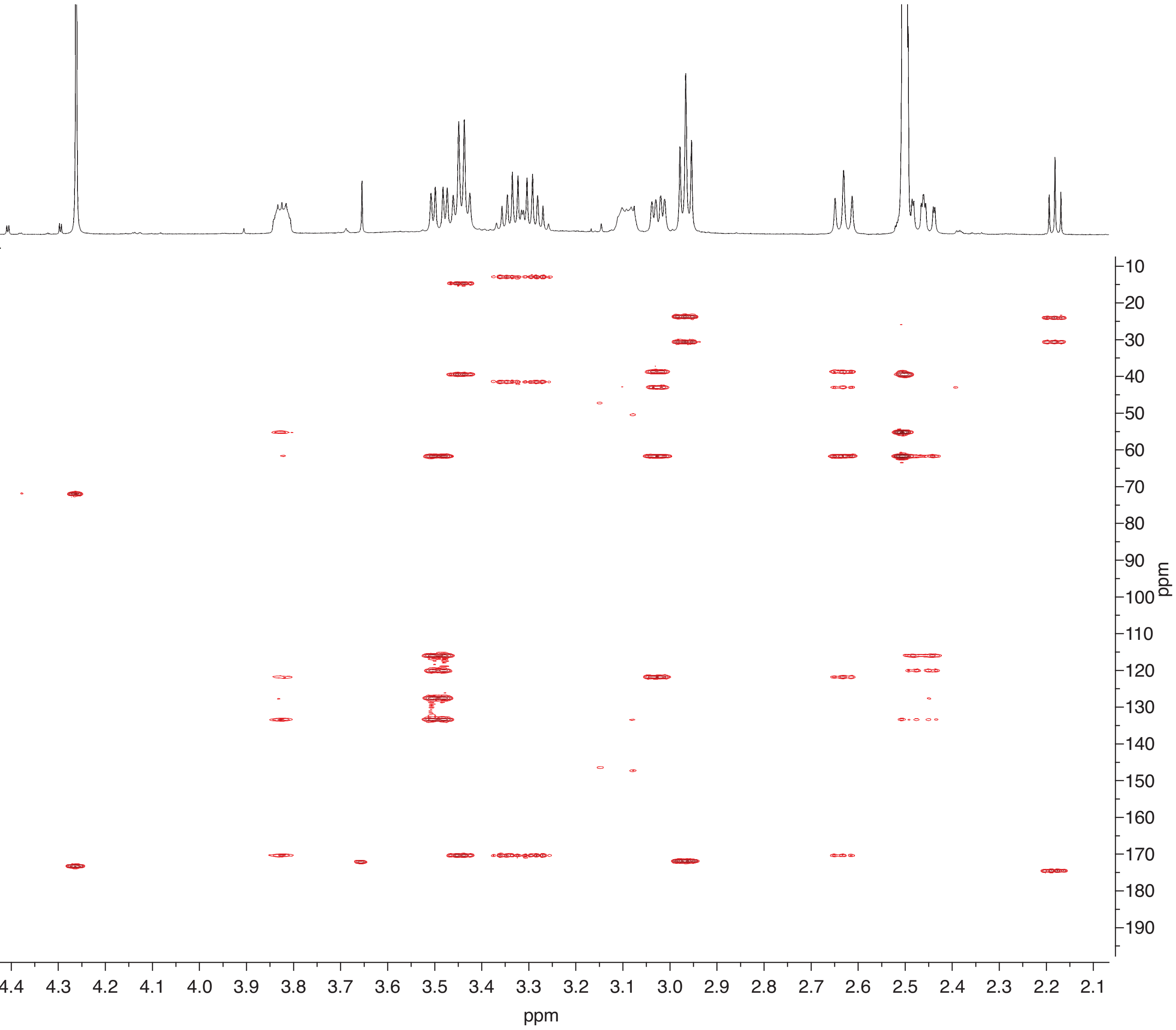

1H-LSD tartrate
HMBC (600/175 MHz)
DMSO-d_6

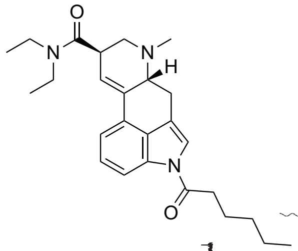

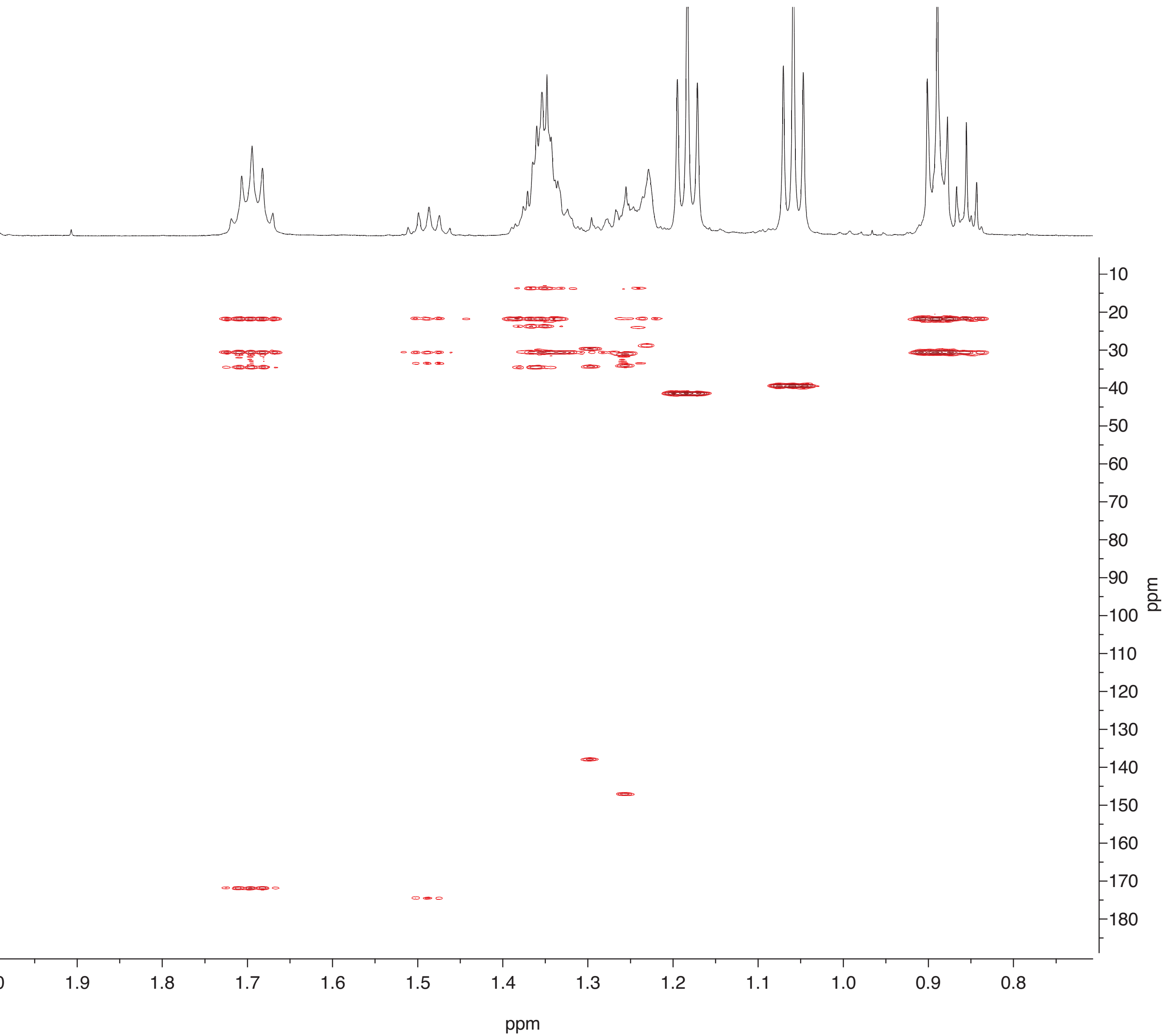

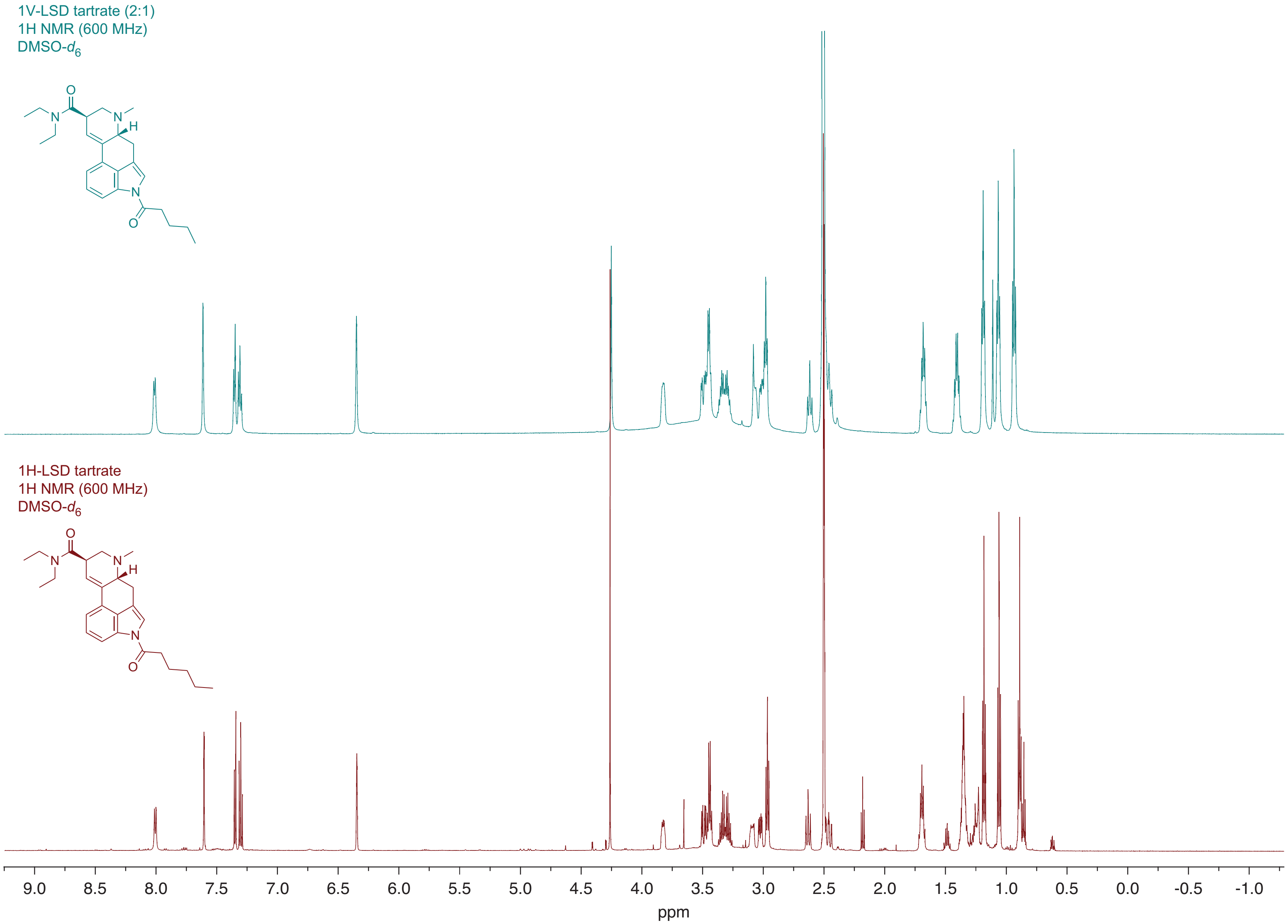
1V-LSD tartrate (2:1)
1H NMR (600 MHz)
DMSO-d6
1H-LSD tartrate
1H NMR (600 MHz)
DMSO-d6
9.0
8.5
8.0
7.5
7.0
6.5
6.0
5.5
5.0
4.5
4.0
3.5
3.0
2.5
2.0
1.5
1.0
0.5
0.0
-0.5
-1.0
ppm

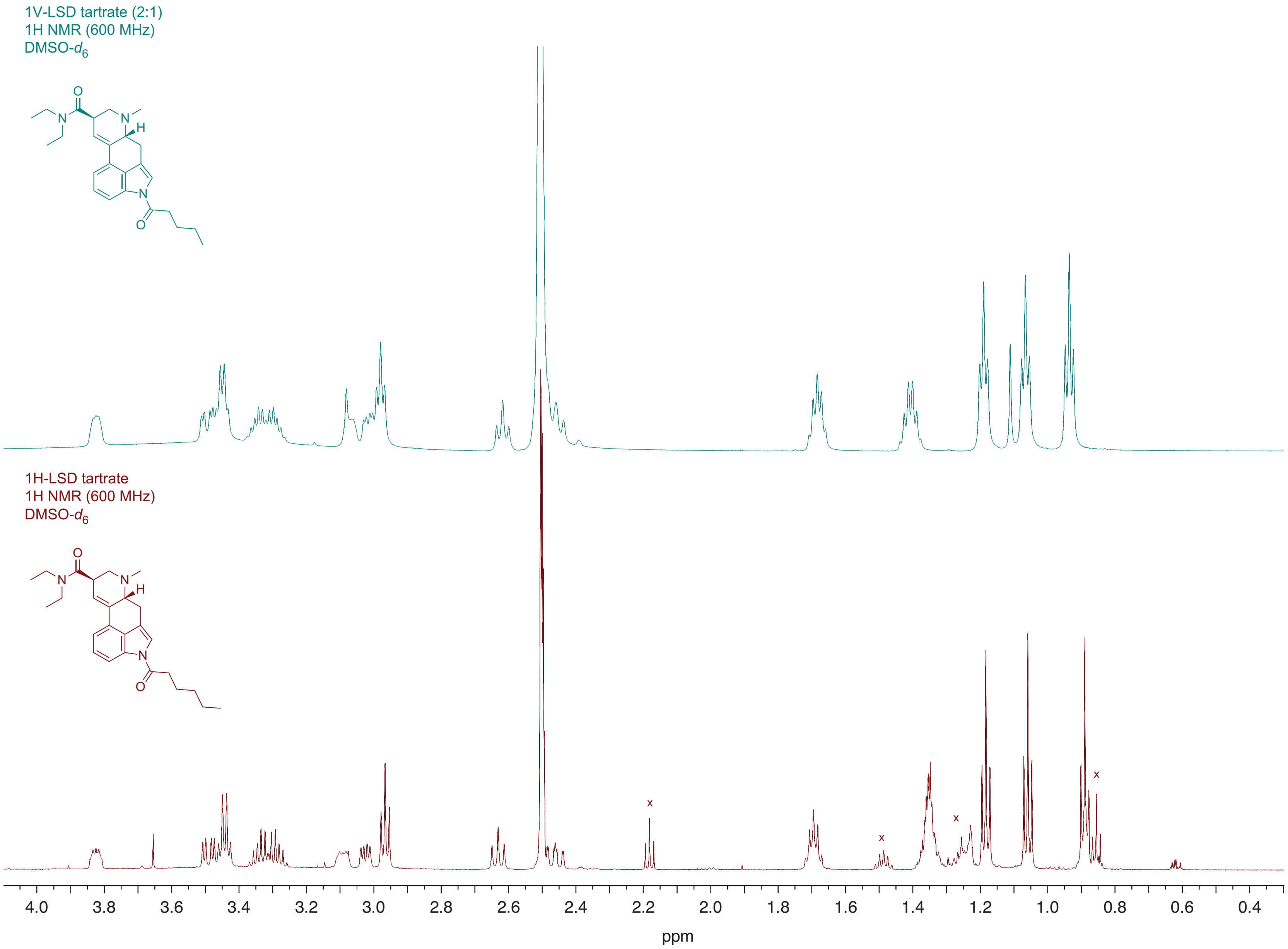

X: Heaxanoic acid

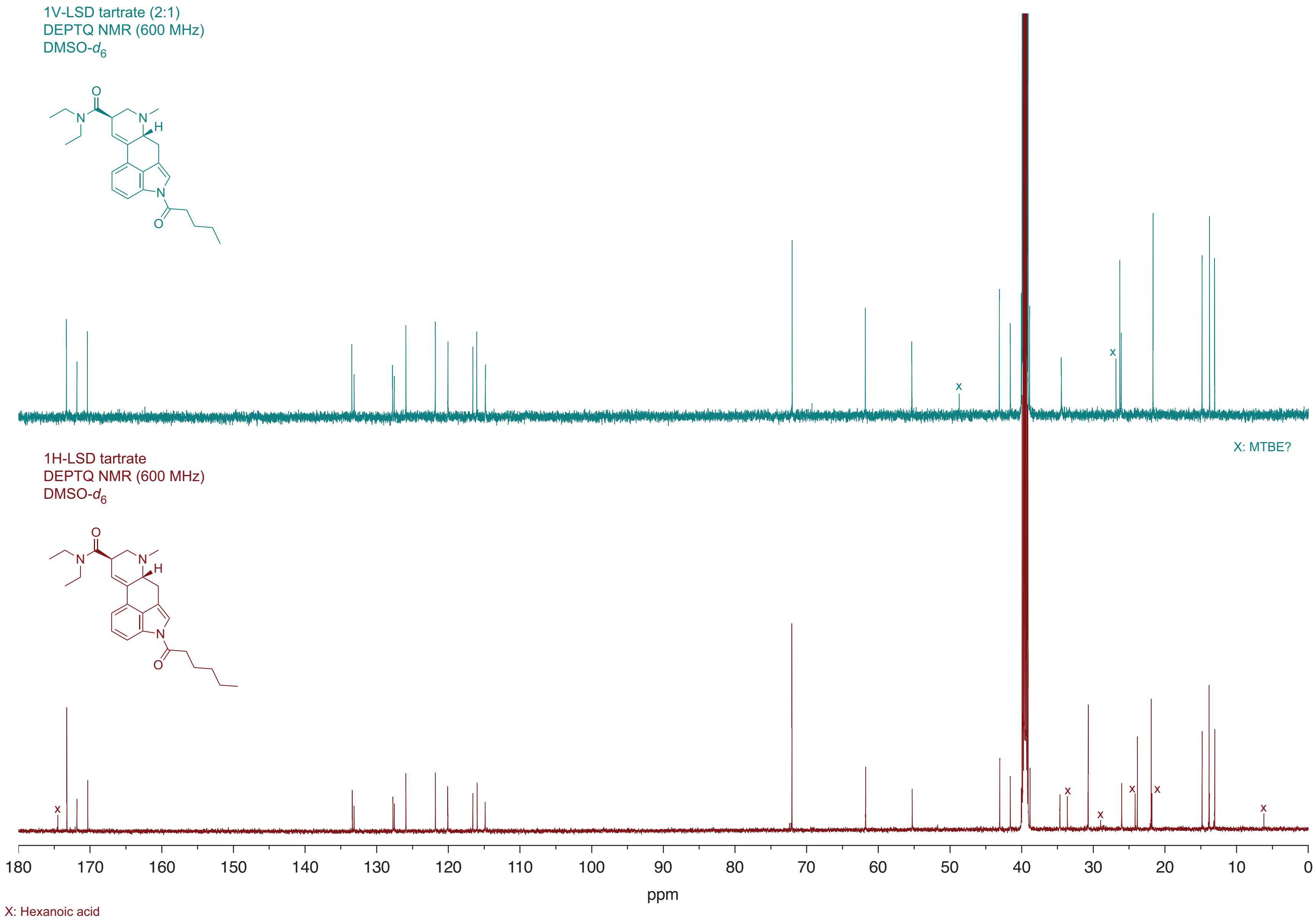

1V-LSD tartrate (2:1)
DEPTQ NMR (600 MHz)
DMSO-d_6
X: MTBE?
1H-LSD tartrate
DEPTQ NMR (600 MHz)
DMSO-d_6
180 170 160 150 140 130 120 110 100 90 80 70 60 50 40 30 20 10 0
ppm
X: Hexanoic acid

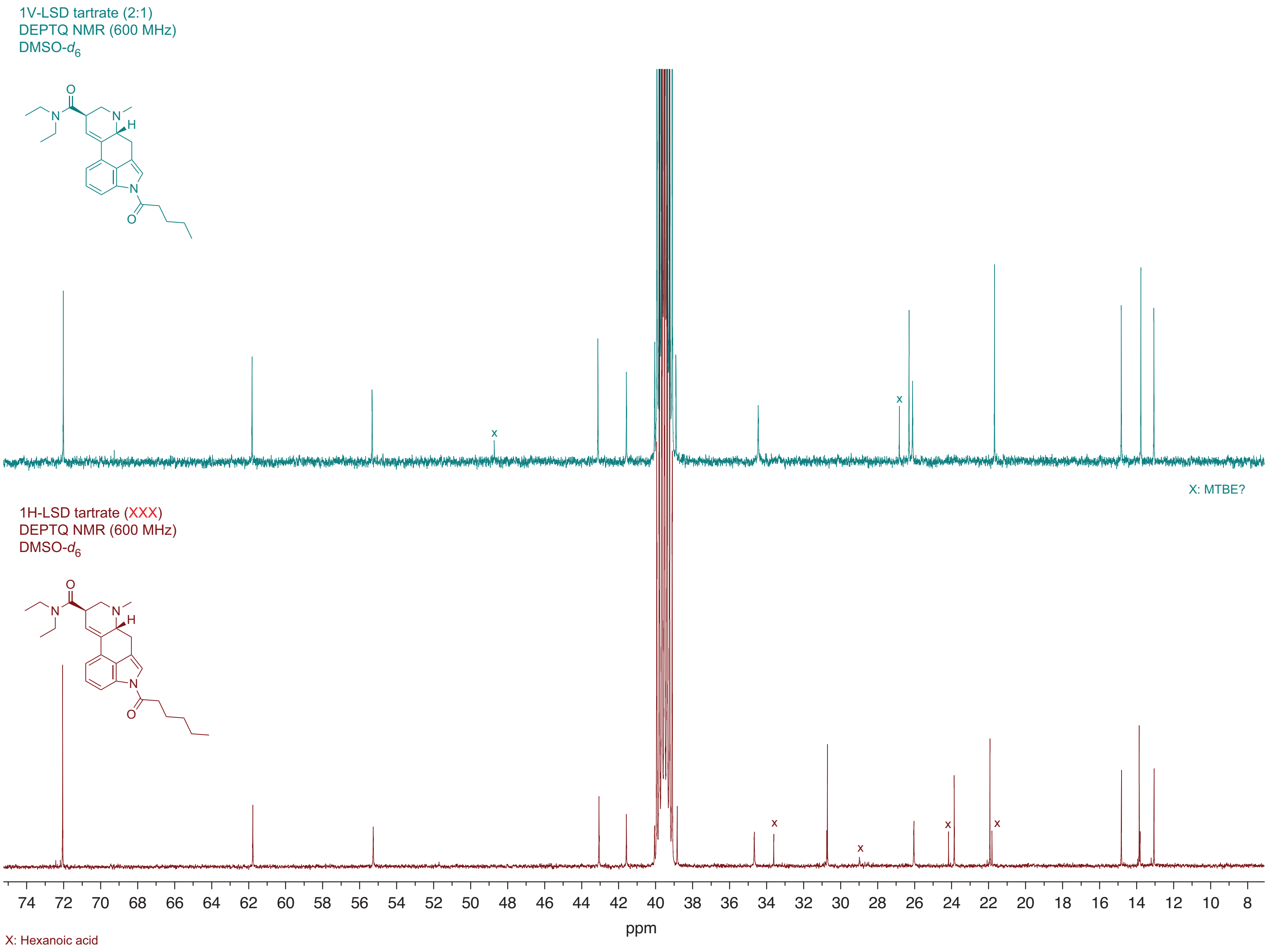
1V-LSD tartrate (2:1)
DEPTQ NMR (600 MHz)
DMSO-d_6
X: MTBE?
1H-LSD tartrate (XXX)
DEPTQ NMR (600 MHz)
DMSO-d_6
74 72 70 68 66 64 62 60 58 56 54 52 50 48 46 44 42 40 38 36 34 32 30 28 26 24 22 20 18 16 14 12 10 8
ppm
X: Hexanoic acid

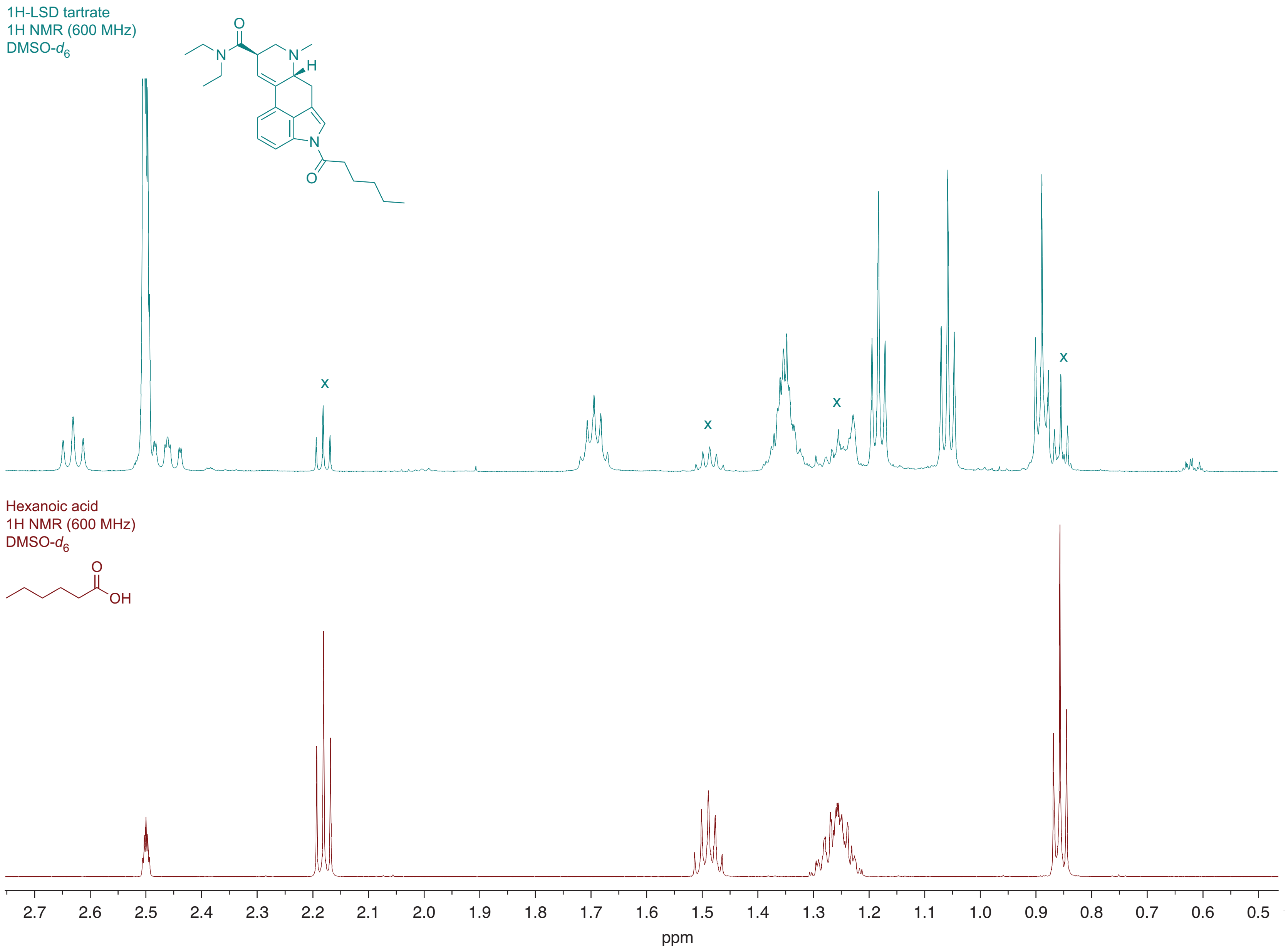
1H-LSD tartrate
1H NMR (600 MHz)
DMSO-d_6
O
N
N
H
N
O
x
x
x
x
Hexanoic acid
1H NMR (600 MHz)
DMSO-d_6
O
OH
2.7
2.6
2.5
2.4
2.3
2.2
2.1
2.0
1.9
1.8
1.7
1.6
1.5
1.4
1.3
1.2
1.1
1.0
0.9
0.8
0.7
0.6
0.5
ppm

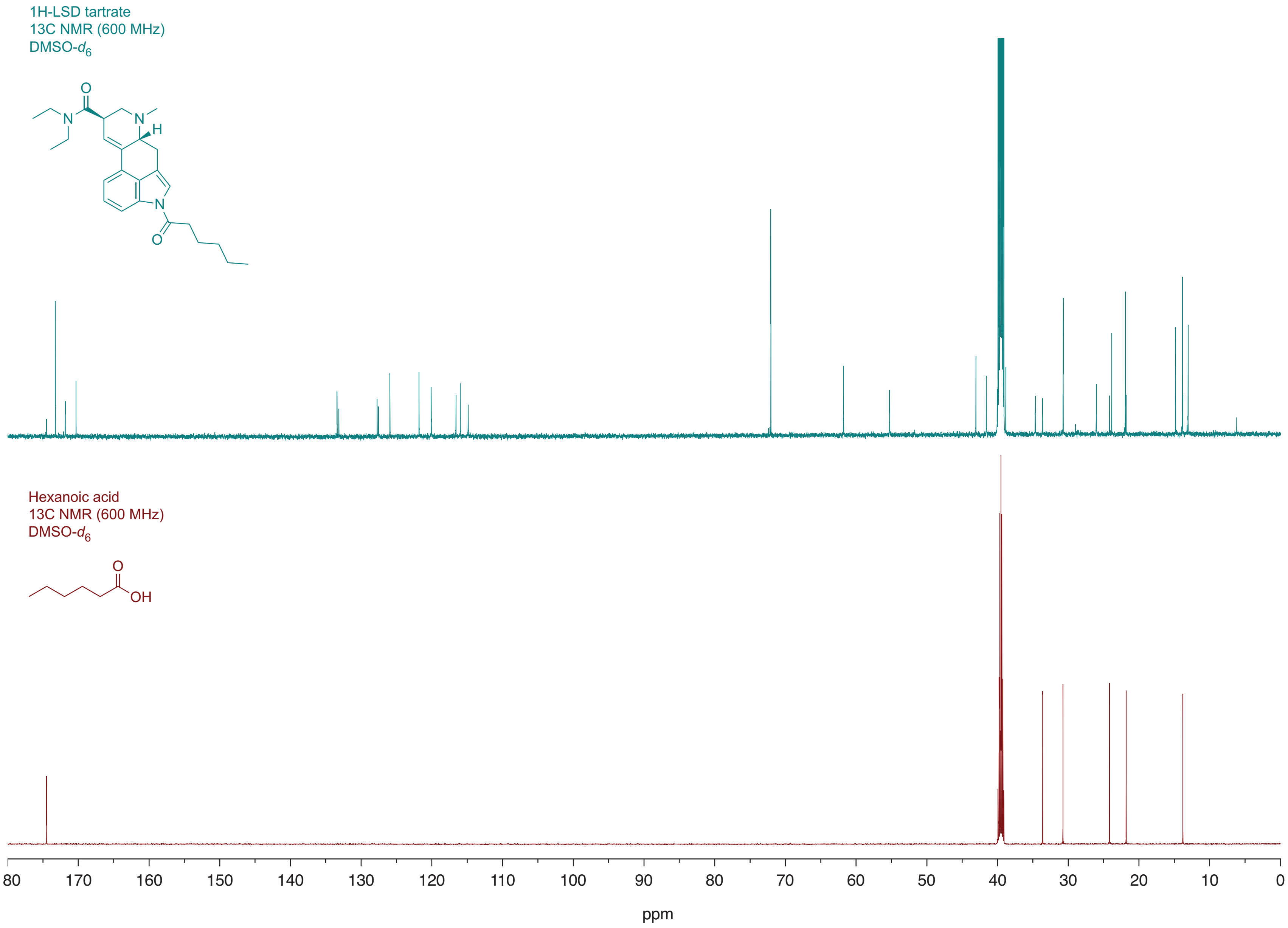
1H-LSD tartrate
13C NMR (600 MHz)
DMSO-d_6
O
N
N
H
N
O
Hexanoic acid
13C NMR (600 MHz)
DMSO-d_6
O
OH
180 170 160 150 140 130 120 110 100 90 80 70 60 50 40 30 20 10 0
ppm

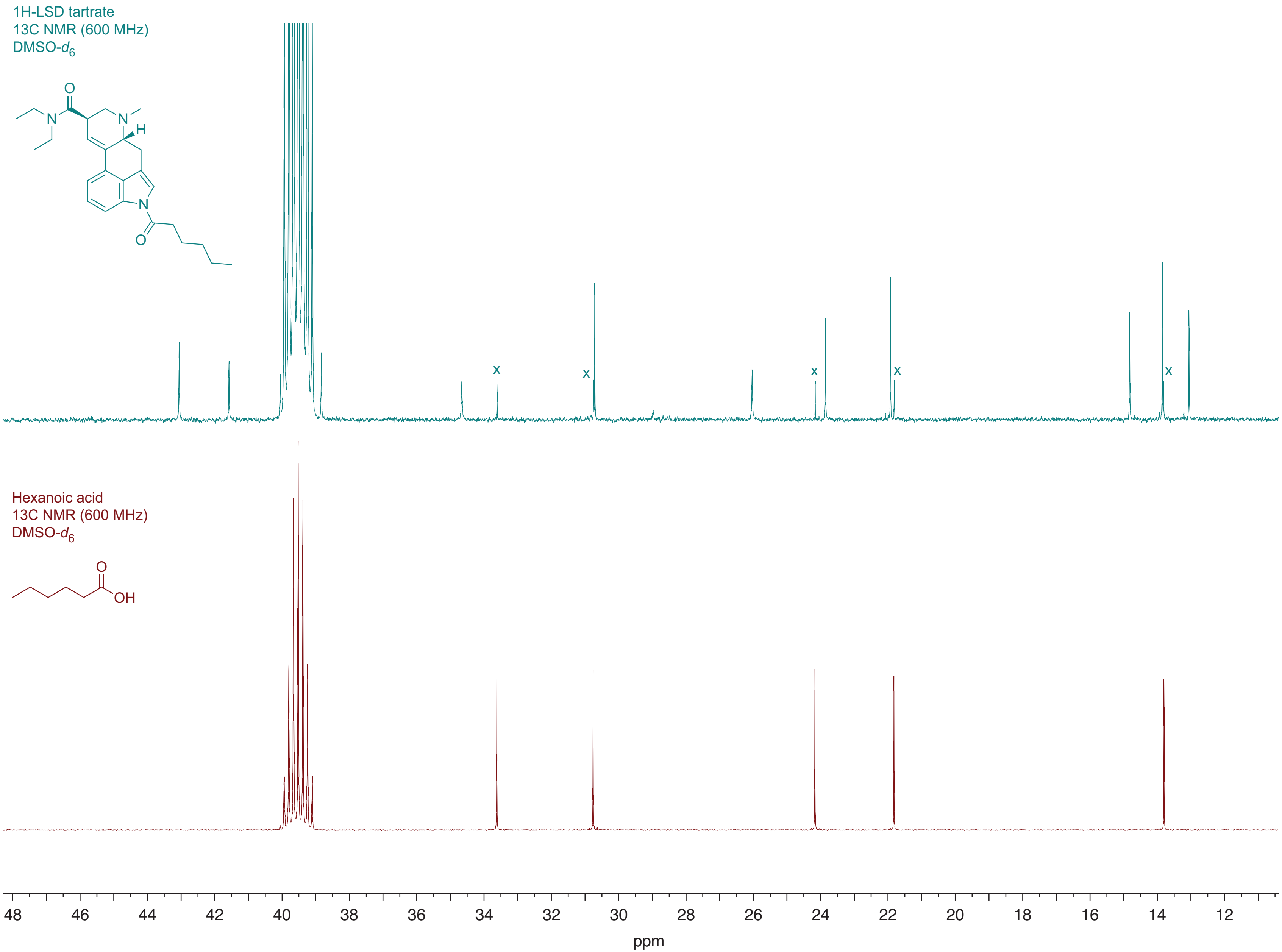
1H-LSD tartrate
13C NMR (600 MHz)
DMSO-d_6
O
N
N
H
N
O
x
x
x
x
x
Hexanoic acid
13C NMR (600 MHz)
DMSO-d_6
O
OH
48
46
44
42
40
38
36
34
32
30
28
26
24
22
20
18
16
14
12
ppm
